# Supplementary figures and images for: Identification of C. elegans ASNA-1 domains and tissue requirements that differentially influence platinum sensitivity and growth control
Source: PLoS Genet. 2022 Dec 8;18(12):e1010538. doi: 10.1371/journal.pgen.1010538 (PMC9803280; doi:10.1371/journal.pgen.1010538)

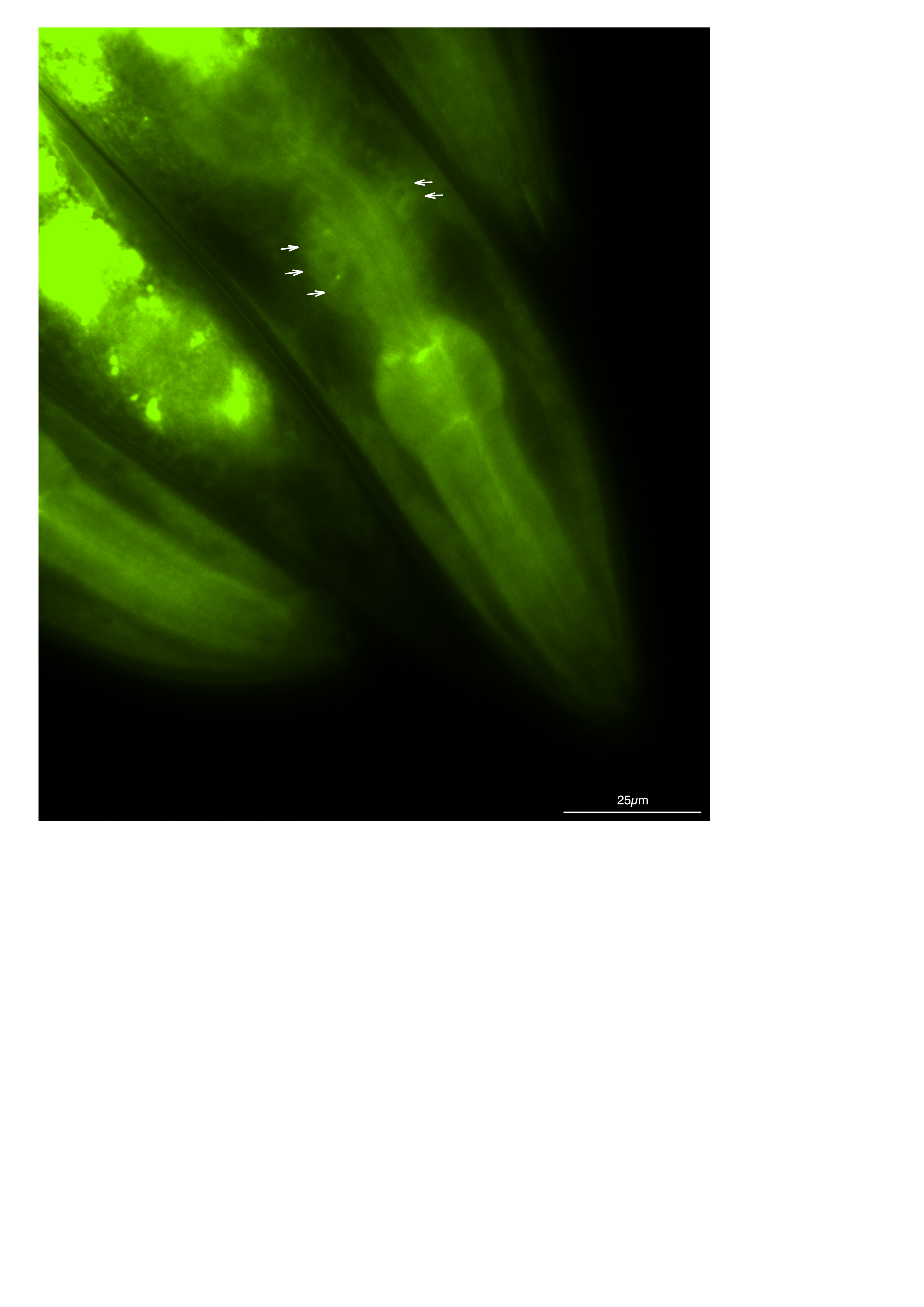

Supplement: S1 Fig — Representative images of 1-day old adult worms expressing ASNA-1::mNG::AID (syb2249) in head neurons (white arrows). (TIFF) [file pgen.1010538.s001.tiff]

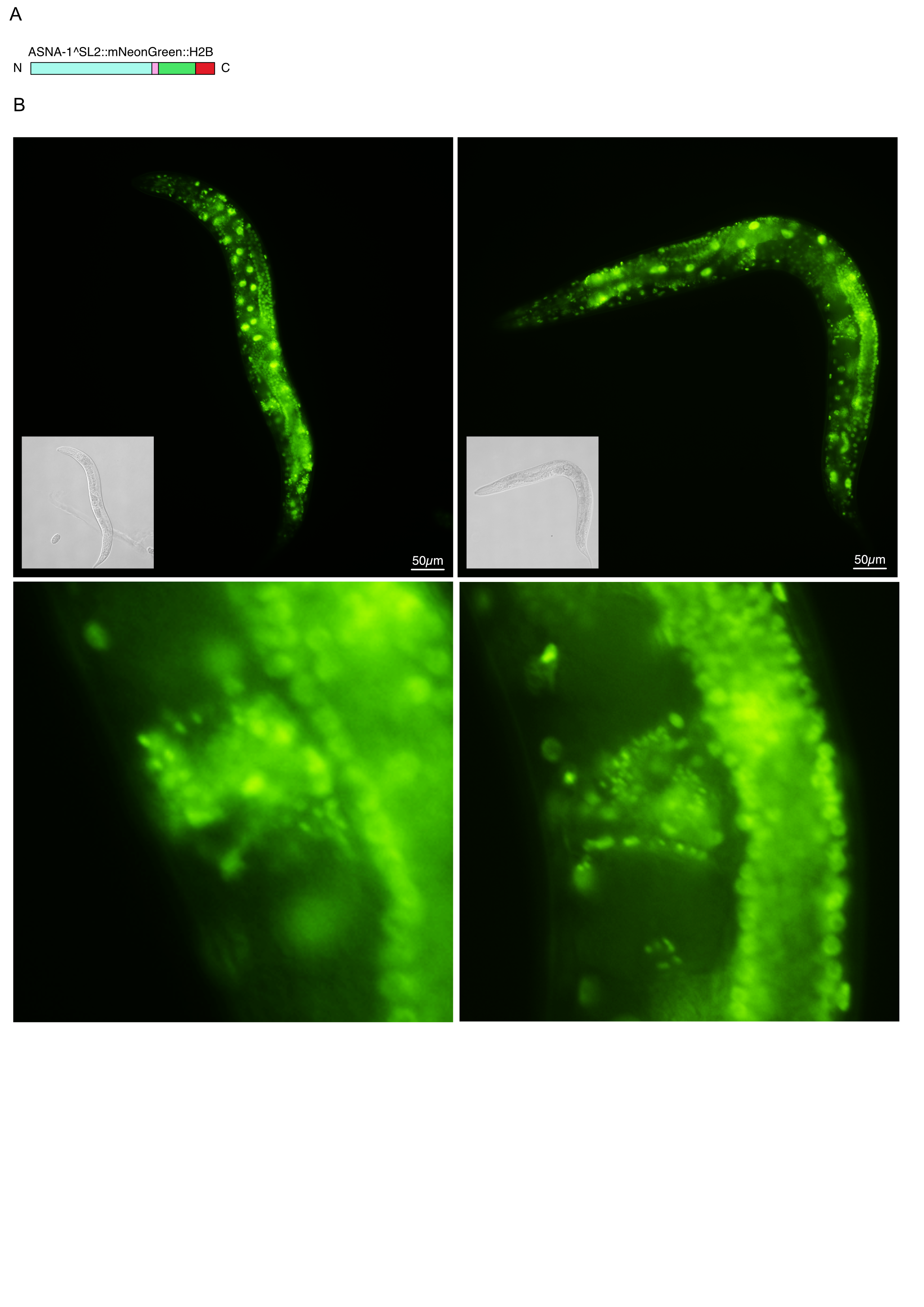

Supplement: S2 Fig — (A) Schematic representation of bi-cistronic ASNA-1^SL2::mNeonGreen::H2B (syb5730). (B) Representative fluorescence and differential interference contrast (DIC) images worms expressing ASNA-1^SL2::mNeonGreen::H2B (syb5730). (TIFF) [file pgen.1010538.s002.tiff]

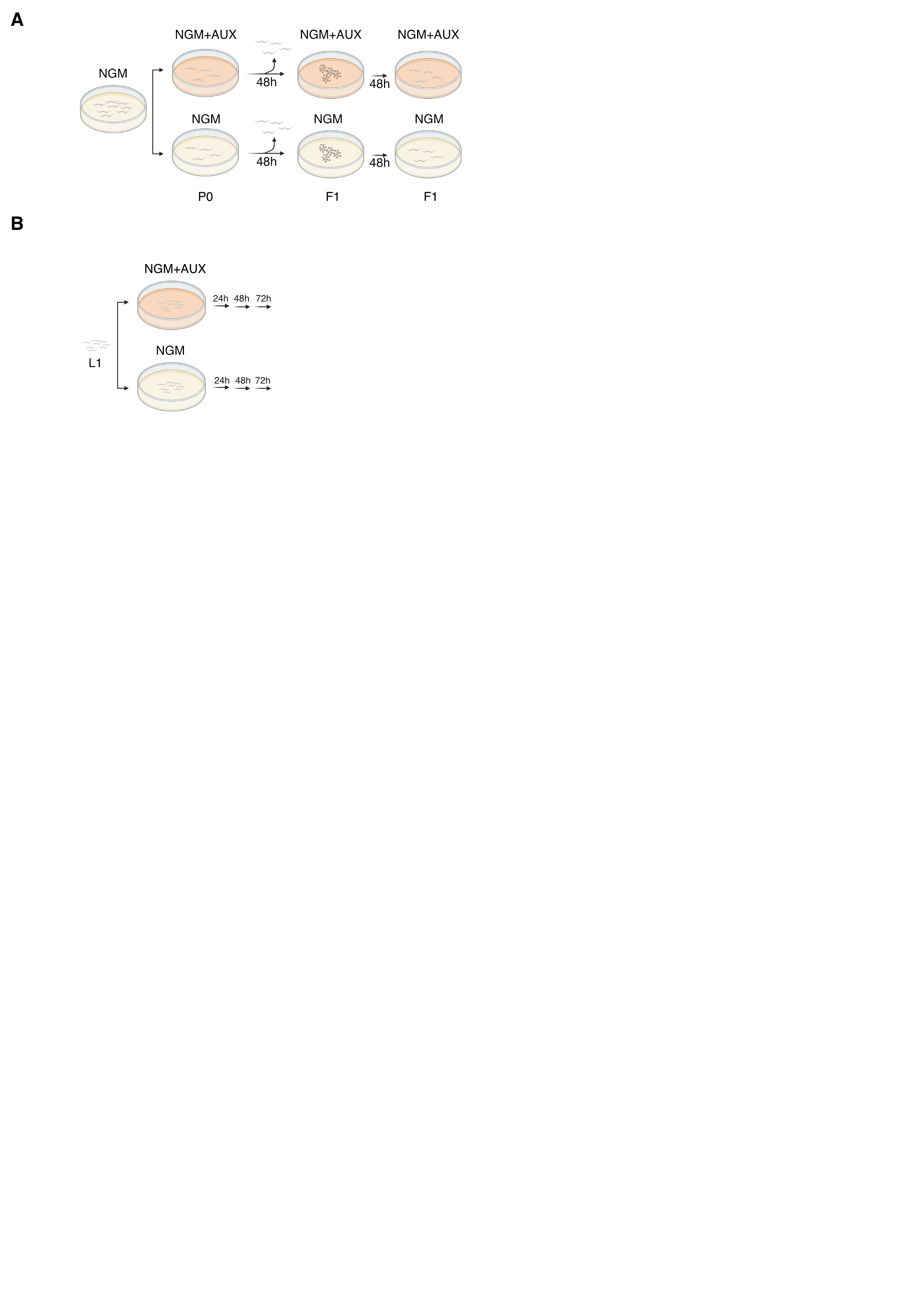

Supplement: S3 Fig — (A) 4th larval stage (L4) hermaphrodites were exposed to 1mM auxin (AUX) for 48h while they produced progeny and then removed from the plate. Their progeny remaining on the auxin-containing plates were analyzed 48 hours after removal of the mothers. Similarly handled L4 worms on non-auxin NGM plates served as a control. (B) Staged L1 hermaphrodites were exposed to 1mM auxin (AUX) for 24h, 48h, and 72h and analyzed at these time points. Similarly staged unexposed larvae on non-auxin NGM plates served as a control. (TIFF) [file pgen.1010538.s003.tiff]

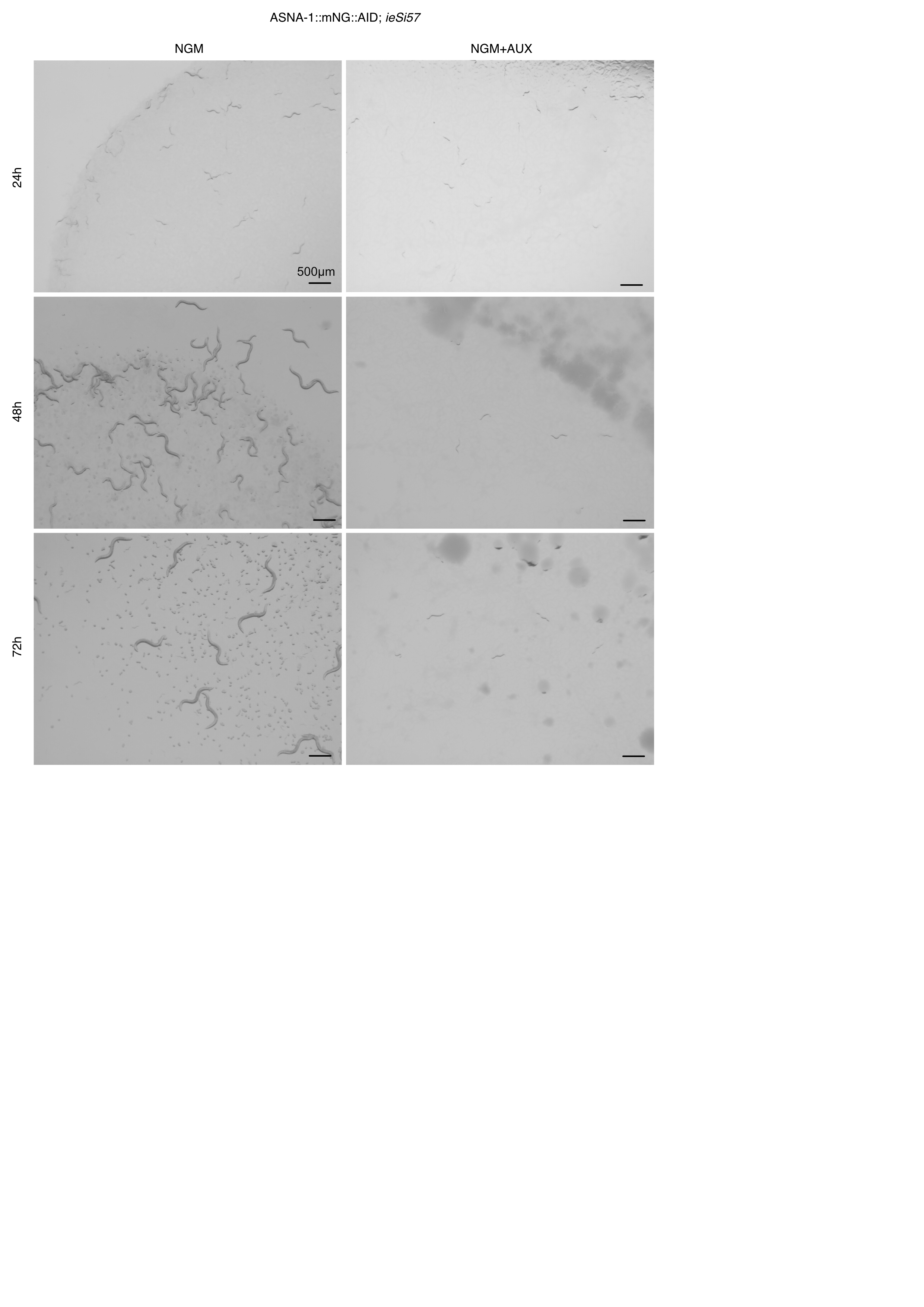

Supplement: S4 Fig — Representative images of worms expressing syb2249; ieSi57 grown on plates without (NGM) or with (NGM+AUX) 1mM auxin at specific timepoints. (TIFF) [file pgen.1010538.s004.tiff]

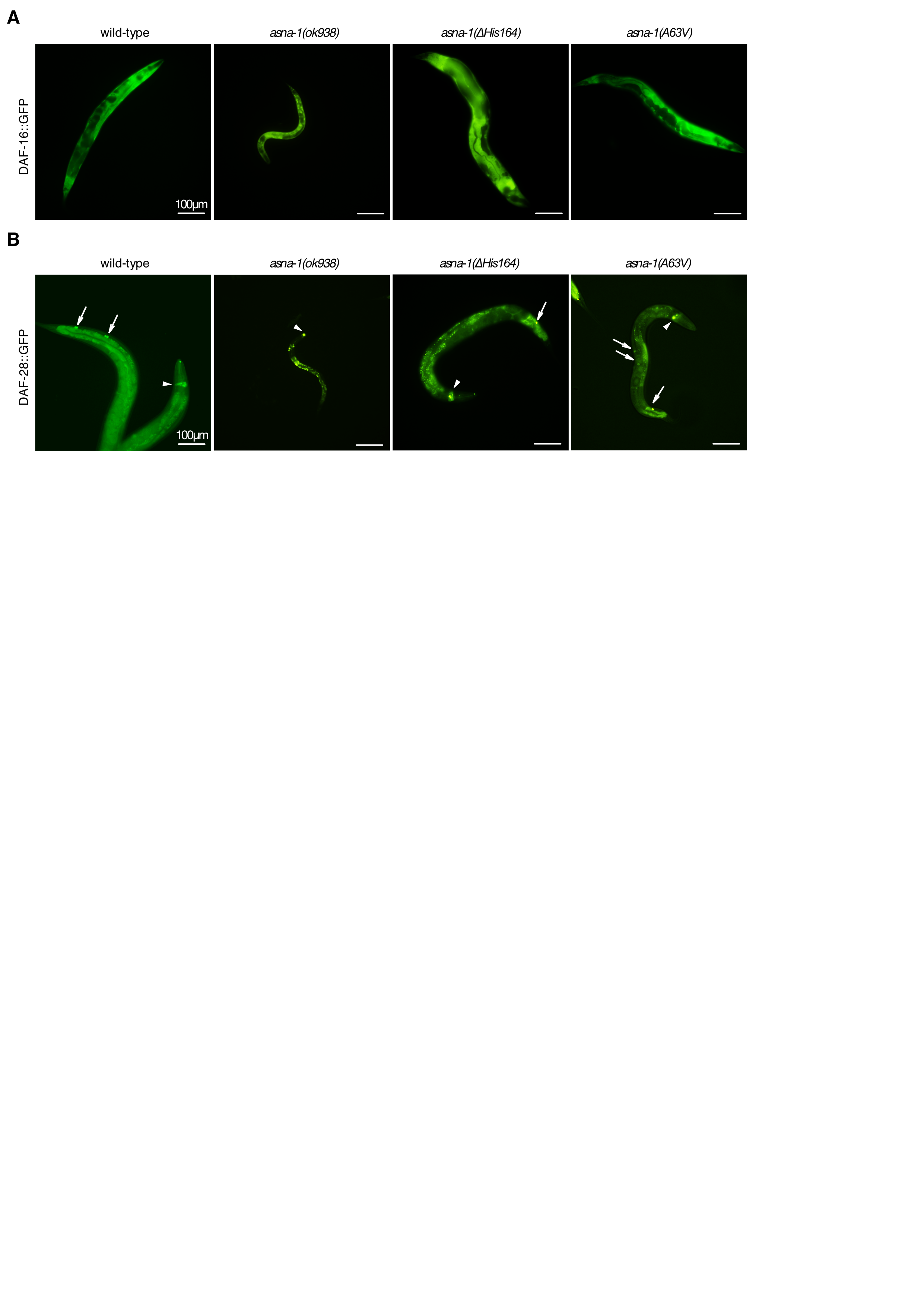

Supplement: S5 Fig — Representative fluorescence images of wild-type, asna-1(ok938), asna-1(ΔHis164) and asna-1(A63V) adult worms expressing (A) DAF-16::GFP or (B) DAF-28::GFP. White arrows indicate coelomocyte expressing DAF-28::GFP and white triangles indicate neurons expressing DAF-28::GFP. (TIFF) [file pgen.1010538.s005.tiff]

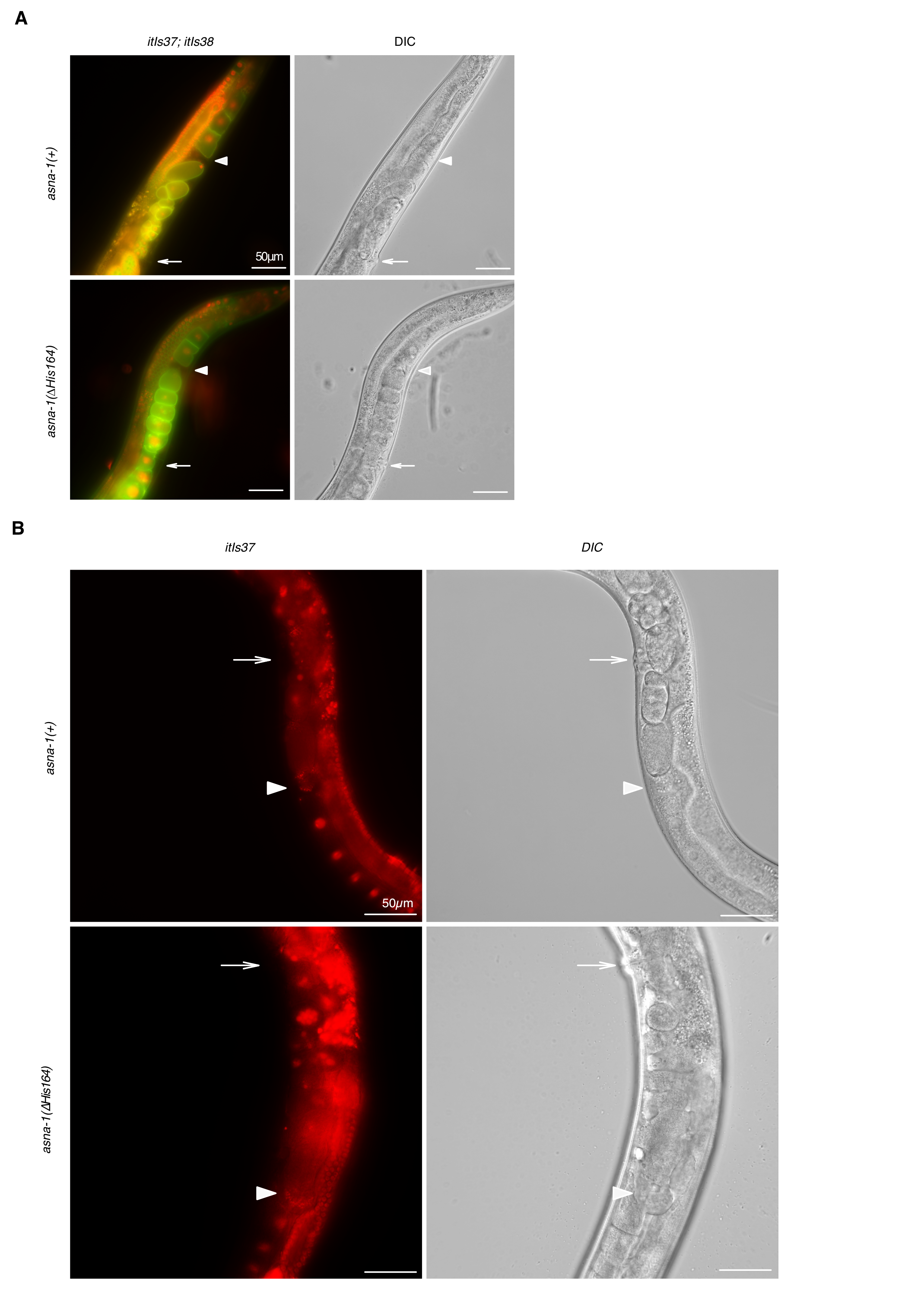

Supplement: S6 Fig — (A) Representative fluorescent and light microscopy pictures of asna-1(+) and asna-1(ΔHis164) adult animals expressing itIs37 transgene to visualize germline nuclei (red) and itIs38 transgene to visualize germ cell membranes (green) in vivo. White arrows indicate vulva location and white triangles indicate spermatheca placement. (B) Representative fluorescent and light microscopy pictures of asna-1(+) and asna-1(ΔHis164) adult animals expressing itIs37 transgene to visualize germline nuclei in vivo. White arrows indicate the location of the vulva for the purposes of orientation and white triangles indicate sperm in the spermatheca. (TIFF) [file pgen.1010538.s006.tiff]

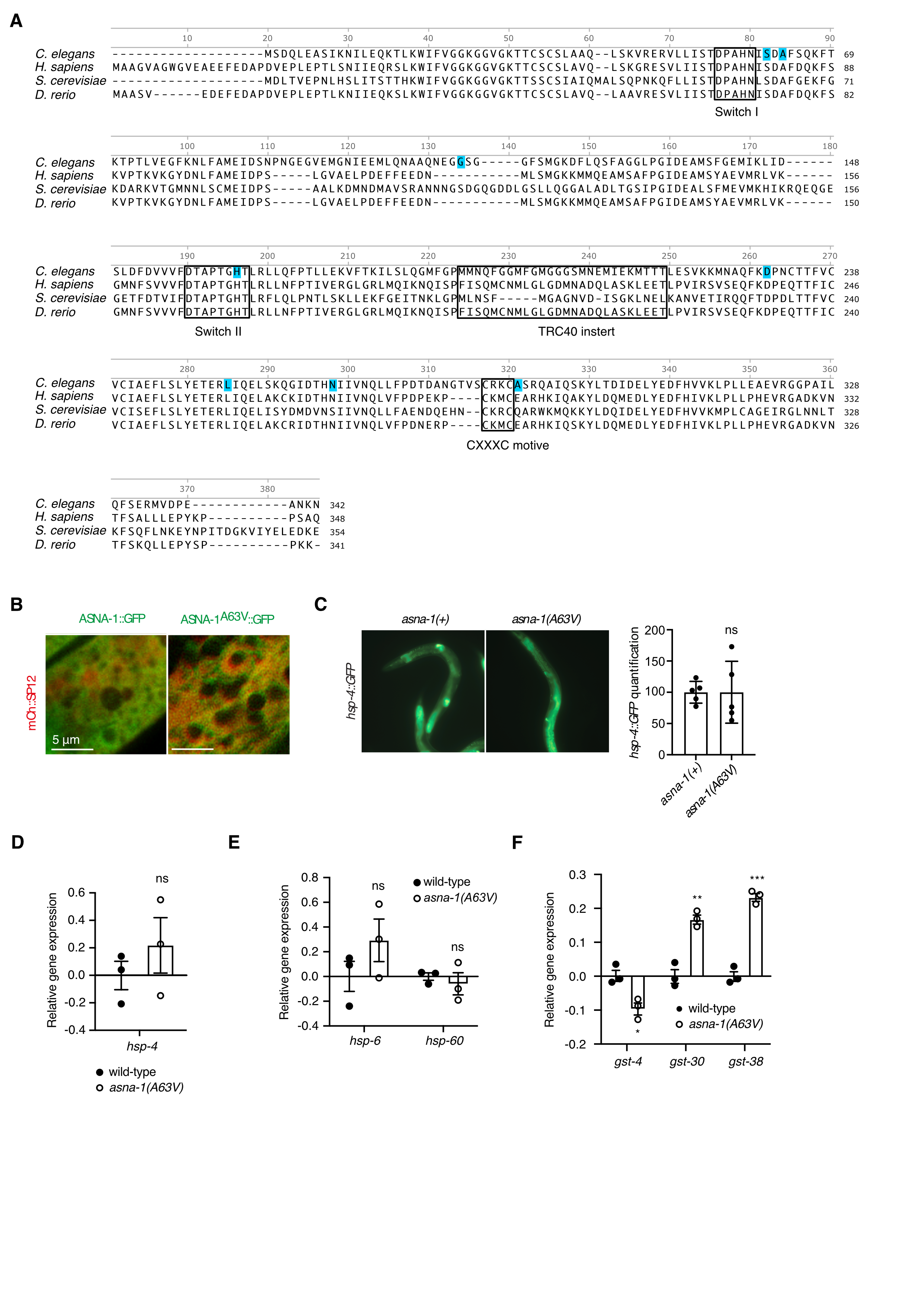

Supplement: S7 Fig — (A) Multiple sequence alignment of ASNA-1/GET3/TRC40 comparing particular domains of the protein in 4 different species. The essential domains are highly conserved. Amino acids mentioned throughout the paper are marked in blue. (B) Confocal imaging merge of 1-day old adults co-expressing mCherry::SP12 with either ASNA-1::GFP or ASNA-1A63V::GFP. (C) Expression from the hsp-4p::GFP reporter (zcIs4) imaged by fluorescence microscopy in the 1-day old adult wild-type and asna-1(A63V) animals. hsp-4p::GFP expression quantification in the wild-type (n = 5) and asna-1(A63V) animals (n = 5). Statistical significance was determined by the independent two-sample t-test. Bars represent mean ± SD. (D) Relative mRNA analysis of ER stress reporter hsp-4 in 1-day old adult asna-1(A63V) animals. Statistical significance was determined by the independent two-sample t-test. Experiments were performed in triplicate. F44B9.5 was used as a normalizing control. Bars represent mean ± SEM. Relative mRNA analysis of (E) the mitochondrial stress reporters (hsp-6 and hsp-60) and (F) oxidative stress reporters (gst-4, gst-30, and gst-38) in 1-day old adult wild-type and asna-1(A63V) animals. Statistical significance was determined by the independent two-sample t-test. Experiments were performed in triplicate. F44B9.5 was used as a normalizing control. Bars represent mean ± SEM. (TIFF) [file pgen.1010538.s007.tiff]

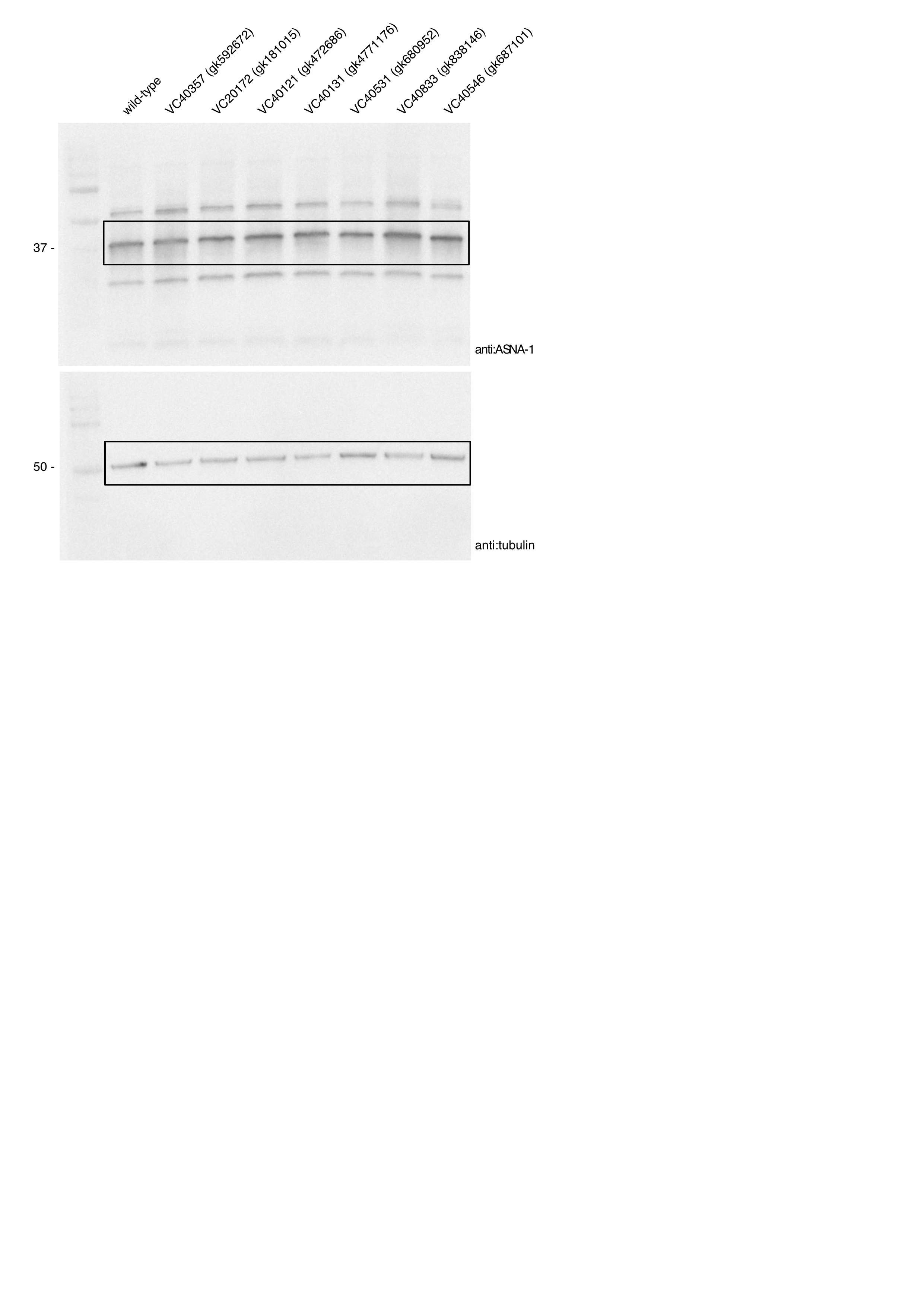

Supplement: S8 Fig — Full uncropped image of Western blot to estimate ASNA-1 levels in animals carrying a single amino acid mutation in ASNA-1. The blot was probed with an anti:ASNA-1 antibody. Tubulin was used as a loading control. (TIFF) [file pgen.1010538.s008.tiff]

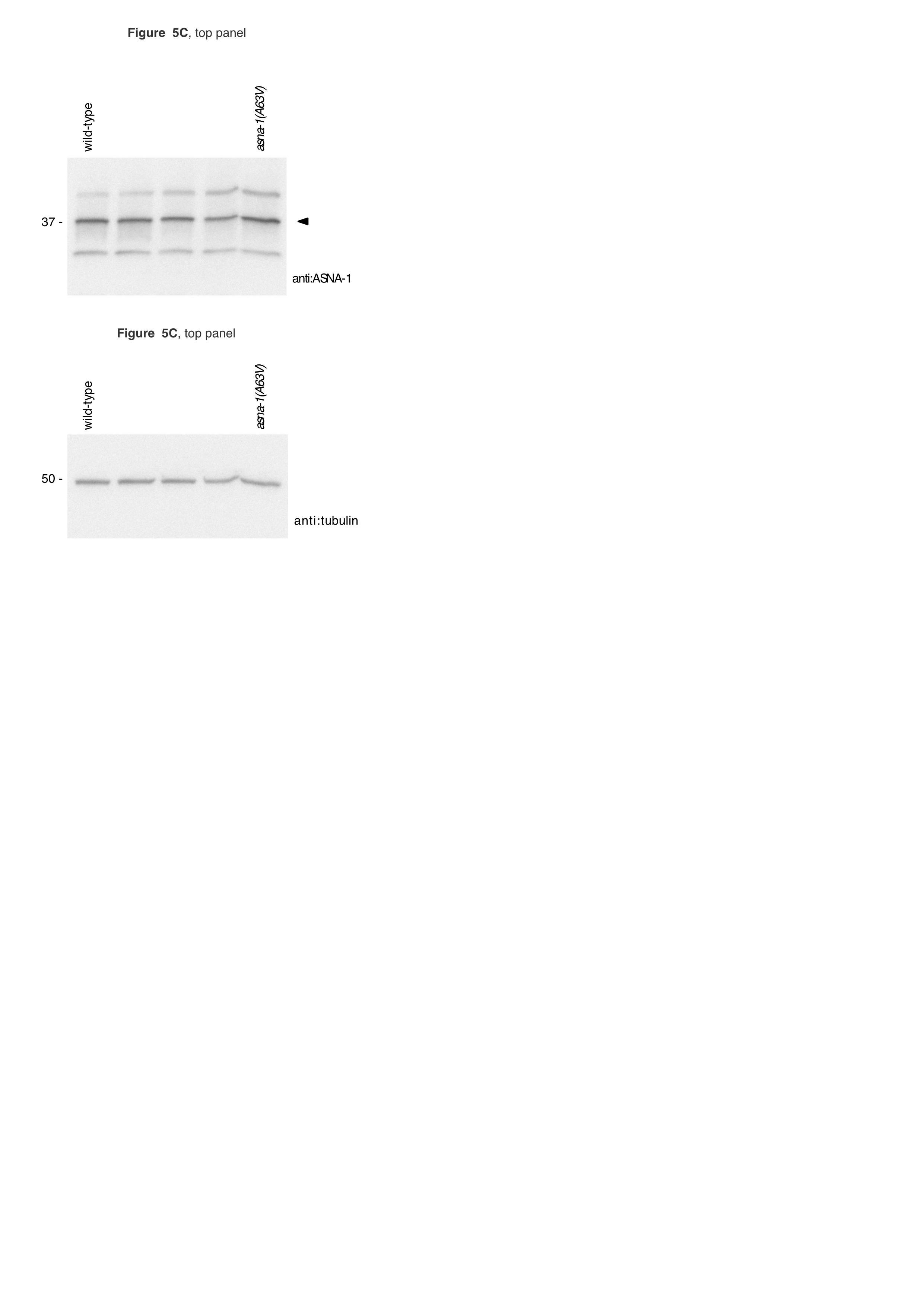

Supplement: S9 Fig — Full uncropped blot image used in Fig 5C. (TIFF) [file pgen.1010538.s009.tiff]

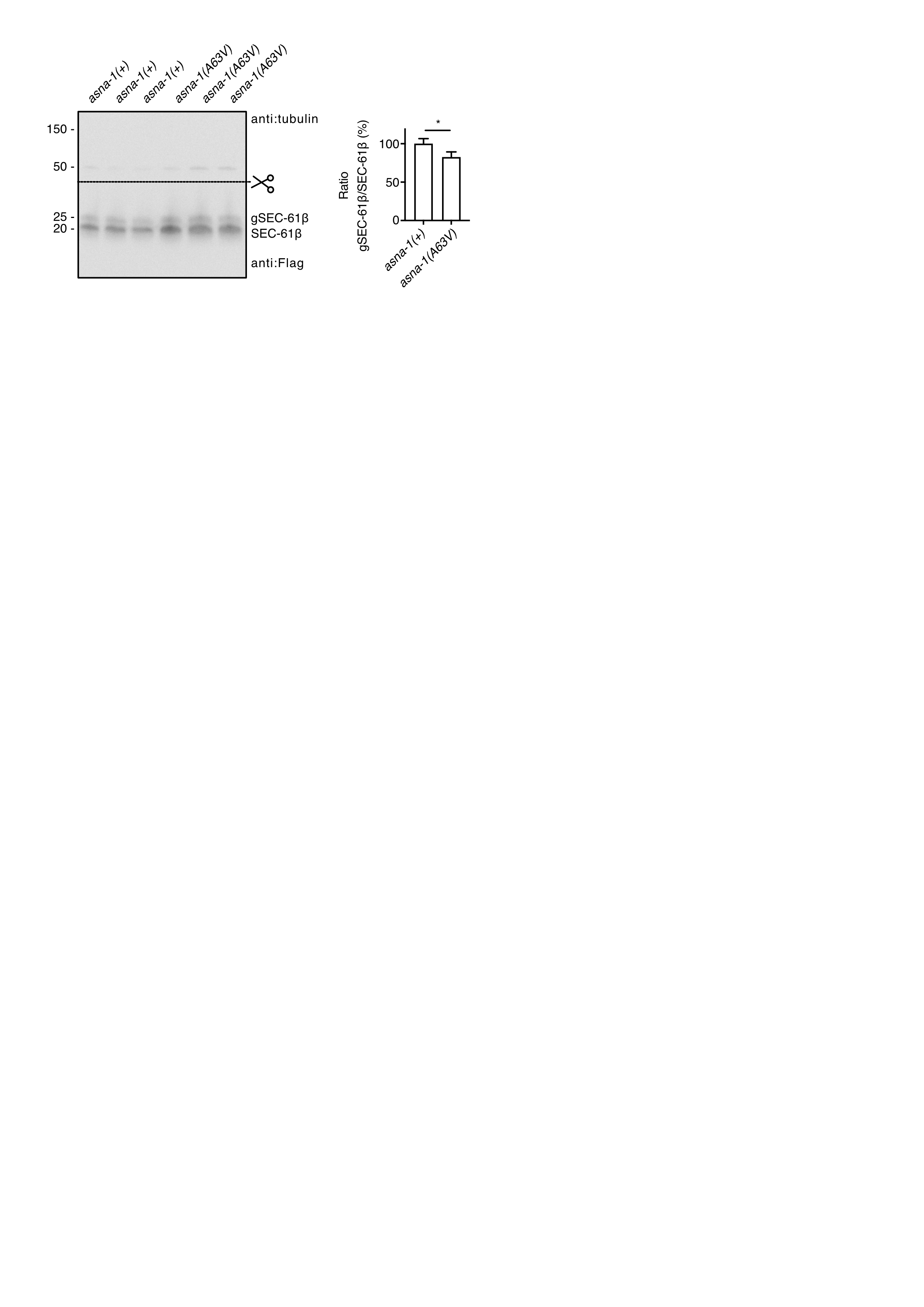

Supplement: S10 Fig — Full uncropped image of Western blot following reducing SDS-PAGE to detect glycosylated (gSEC-61β) and non-glycosylated (SEC-61β) SEC-61β in strains carrying 3xFlag::SEC-61β::opsin (rawEx64) transgene in asna-1(+) and asna-1(A63V) background. The dotted line represents a place of membrane cut in order to simultaneously probe with anti:Flag and anti:tubulin antibodies. Band intensity quantification of glycosylated vs non-glycosylated SEC-61β (gSEC-61β/ SEC-61β). Bars represent mean ± SD. Statistical significance was determined by the independent two-sample t-test. (TIFF) [file pgen.1010538.s010.tiff]

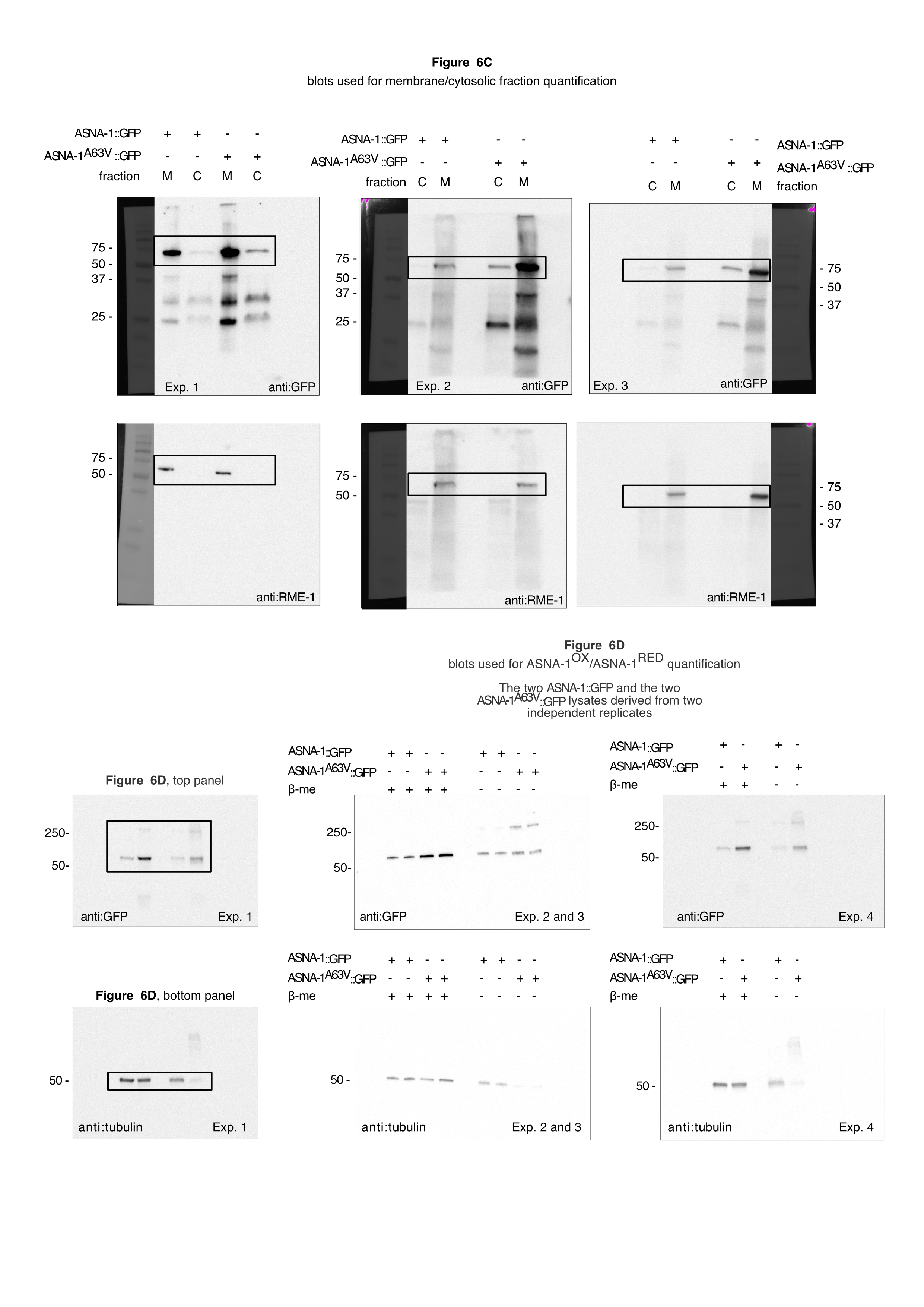

Supplement: S11 Fig — Full uncropped blot image used for quantification in Fig 6C and 6D. (TIFF) [file pgen.1010538.s011.tiff]

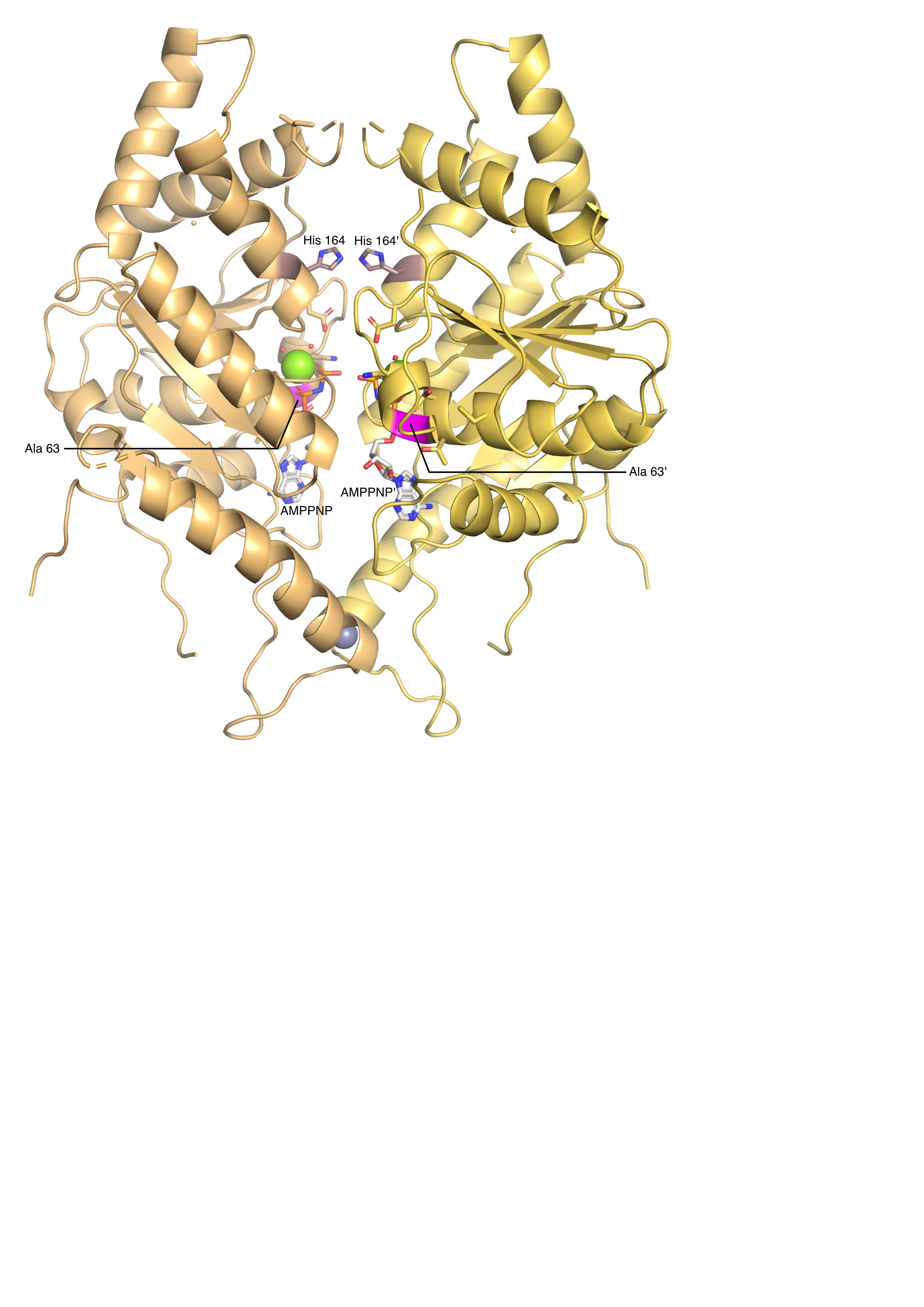

Supplement: S12 Fig — Ribbon diagram of the ASNA-1 dimer obtained with MODELLER in its “closed conformation”, bound to AMPPNP and mimicking the configuration of the Get3 protein complex of Chaeromium thermophilum (PDB:3IQW). The peptide chains are represented in light-orange and yellow-orange, in white the AMPPNP, in green Mg atom, in grey Zn atom, in pink the Ala63, in deep salmon the His164. (TIFF) [file pgen.1010538.s012.tiff]

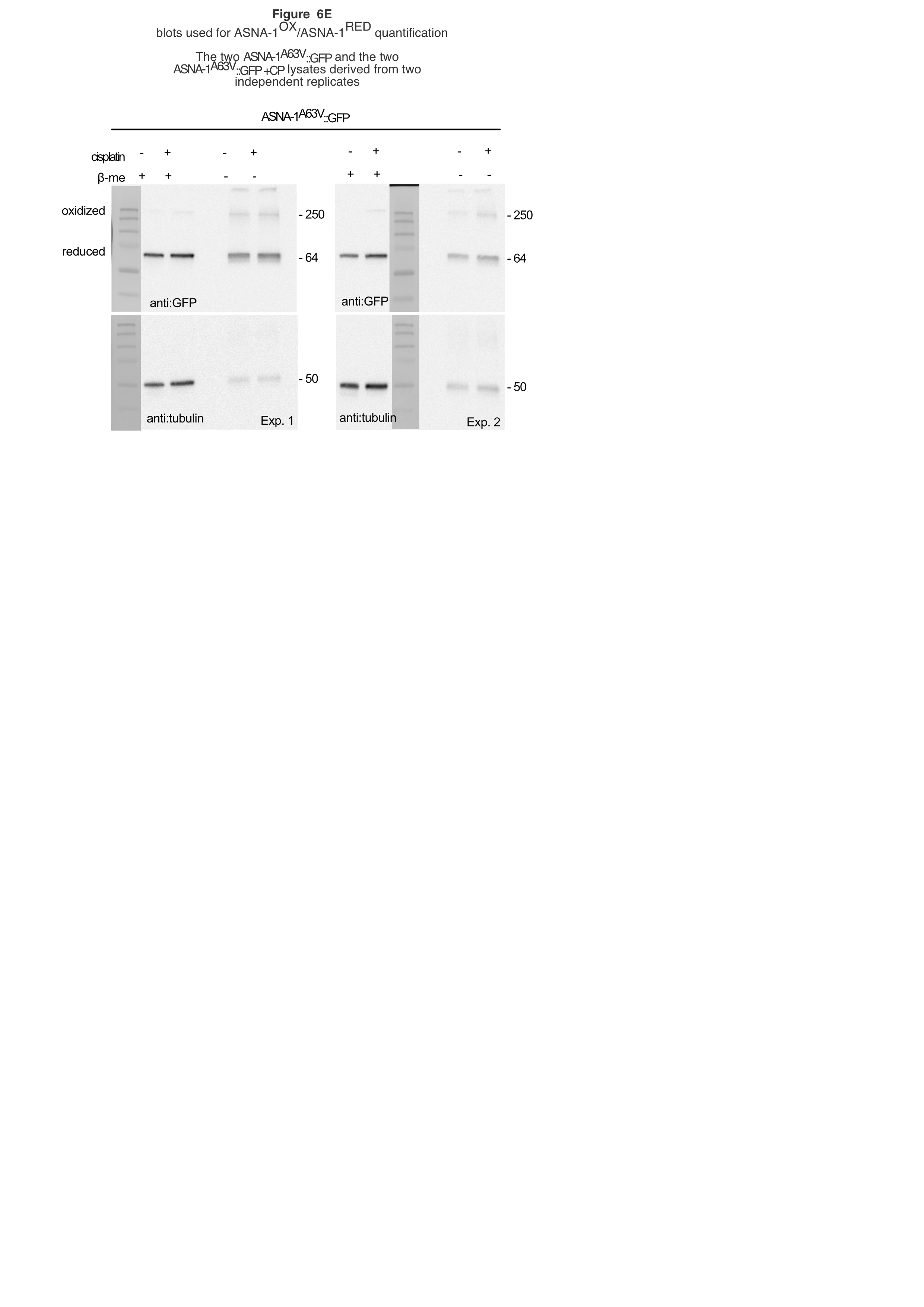

Supplement: S13 Fig — Full uncropped blot image used for quantification in Fig 6E. (TIFF) [file pgen.1010538.s013.tiff]

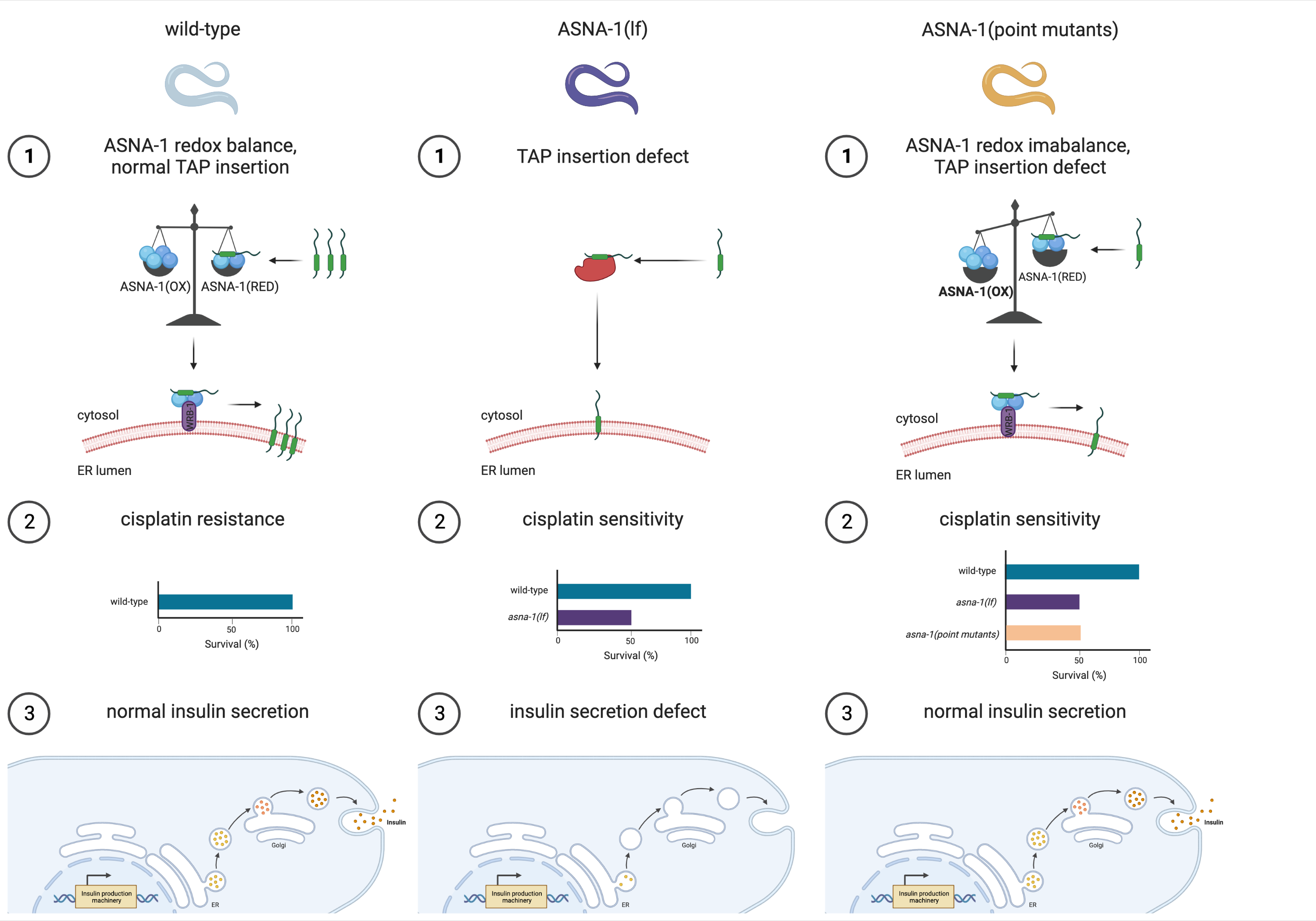

Supplement: S14 Fig — Schematic model to describe function separation of the ASNA-1 protein showing the effects on redox balance (1), cisplatin resistance/sensitivity (2) and DAF-28/insulin secretion (3). A tail anchored protein (TAP) is shown in green with a cylindrical C-terminal membrane spanning domain. Normal TAP insertion is depicted with three TAP protein molecules in the ER membrane bilayer, while lower TAP insertion is shown with one such molecule. ASNA-1 protein in loss-of-function (lf) scenario is shown in red to indicate the low ASNA-1 levels that are present only because of maternal inheritance in the homozygous mutants from asna-1/+ mothers. The altered redox balance in the point mutants (A63V and ΔHis164) resulting in lower levels of reduced ASNA-1 leads to engagement of fewer TAP protein molecules and consequently fewer TAP protein molecules inserted into the ER membrane bilayer. Created with BioRender. (TIFF) [file pgen.1010538.s014.tiff]
