# Supplementary material for: Identification of C. elegans ASNA-1 domains and tissue requirements that differentially influence platinum sensitivity and growth control
Source: PLoS Genet. 2022 Dec 8;18(12):e1010538. doi: 10.1371/journal.pgen.1010538 (PMC9803280; doi:10.1371/journal.pgen.1010538)
Supplement: S1 Data — (DOCX) [file pgen.1010538.s017.docx]

**Identification of *C. elegans* ASNA-1 domains and tissue requirements that differentially influence platinum sensitivity and growth control**

Dorota Raj^1^, Agnieszka Podraza-Farhanieh^1^, Pablo Gallego^2,#^, Gautam Kao^1,^* and Peter Naredi,^1,3,^*

^1^Department of Surgery, Institute of Clinical Sciences, Sahlgrenska Academy, University of Gothenburg, SE413 45 Gothenburg, Sweden

^2^Department of Medical Biochemistry and Cell Biology, University of Gothenburg, SE405 30 Gothenburg, Sweden

^3^Department of Surgery, Sahlgrenska University Hospital, SE413 45 Gothenburg, Sweden

***Corresponding authors:** [peter.naredi@gu.se](mailto:peter.naredi@gu.se); [gautam.kao@gu.se](mailto:gautam.kao@gu.se)

^#^Current address: Discovery Sciences, BioPharmaceuticals R&D, AstraZeneca, Gothenburg, Sweden

**Fig 2A.** Soma-specific ASNA-1 depletion leads to L1 larval arrest. Graph represents quantification of *syb2249; ieSi57* animals population grown on plates without (NGM) or with (NGM+AUX) 1mM auxin.

|  | **syb2249; ieSi57** | |
| --- | --- | --- |
|  | **- AUX** | **+1mM AUX** |
| **L1** | 0 | 109 |
| **L4** | 104 | 0 |
| **Adult** | 115 | 0 |
| **Total number** | 219 | 109 |
|  |  |  |
| **L1 (%)** | 0 | 100 |
| **L4 (%)** | 47 | 0 |
| **Adult (%)** | 53 | 0 |

**Fig 2B**. Soma-specific ASNA-1 depletion leads to L1 larval arrest. Graph represents 72h time point quantification of *syb2249; ieSi57* animals population grown on plates without (NGM) or with (NGM+AUX) 1mM auxin.

|  | **syb2249; ieSi57** | |
| --- | --- | --- |
|  | **- AUX** | **+1mM AUX** |
| **L1** | 0 | 62 |
| **L4** | 25 | 0 |
| **Adult** | 192 | 4 |
| **Total number** | 217 | 66 |
|  |  |  |
| **L1 (%)** | 0 | 94 |
| **L4 (%)** | 12 | 0 |
| **Adult (%)** | 88 | 6 |

|  | ***syb2249; ieSi57*** | |
| --- | --- | --- |
|  | **- AUX** | **+1mM AUX** |
| **L1** | 0 | 62 |
| **L4** | 25 | 0 |
| **Adult** | 192 | 4 |
| **Total number** | 217 | 66 |
|  |  |  |
| **L1 (%)** | 0 | 94 |
| **L4 (%)** | 12 | 0 |
| **Adult (%)** | 88 | 6 |

**Fig 3**A. Intestine-specific ASNA-1 depletion leads to a developmental delay. Graph represents 72h time point quantification of *syb2249; ieSi57* animals population grown on plates without (NGM) or with (NGM+AUX) 1mM auxin.

|  | ***syb2249; ieSi61*** | |
| --- | --- | --- |
|  | **- AUX** | **+1mM AUX** |
| **L2/L3** | 0 | 12 |
| **L4** | 160 | 50 |
| **Adult** | 480 | 6 |
| **Total number** | 640 | 68 |
|  |  |  |
| **L2/L3 (%)** | 0 | 18 |
| **L4 (%)** | 25 | 74 |
| **Adult (%)** | 75 | 8 |

|  | ***syb2249; ieSi61*** | |
| --- | --- | --- |
|  | **- AUX** | **+1mM AUX** |
| **L2/L3** | 0 | 12 |
| **L4** | 160 | 50 |
| **Adult** | 480 | 6 |
| **Total number** | 640 | 68 |
|  |  |  |
| **L2/L3 (%)** | 0 | 18 |
| **L4 (%)** | 25 | 74 |
| **Adult (%)** | 75 | 8 |

**Fig 4A**. Comparison of germline and insulin/IIS defects in *asna-1(deltaHis164)* and *asna-1(ok938)* deletion mutants. Percentage of 1-day old adults with indicated genotypes displaying only cytoplasmic localized DAF-16::GFP. Experiments were performed in triplicate. Statistical significance was determined by the one-way ANOVA followed by Bonferroni post-hoc correction. Bars represent mean ± SD.

|  | **total number of animals** | **no of animals with cytoplasmic DAF-16::GFP** | **no of animals with nuclear DAF-16::GFP** | **% of animals with cytoplasmic DAF-16::GFP** |
| --- | --- | --- | --- | --- |
| **wild-type** | 15 | 13 | 2 | 87 |
|  | 10 | 10 | 0 | 100 |
|  | 16 | 15 | 1 | 94 |
| ***asna-1(ok938)*** | 10 | 2 | 8 | 20 |
|  | 15 | 4 | 11 | 27 |
|  | 12 | 5 | 7 | 42 |
| ***asna-1(deltaHis164)*** | 15 | 15 | 0 | 100 |
|  | 12 | 12 | 0 | 100 |
|  | 11 | 11 | 0 | 100 |

|  | **total number of animals** | **no of animals with cytoplasmic DAF-16::GFP** | **no of animals with nuclear DAF-16::GFP** | **% of animals with cytoplasmic DAF-16::GFP** |
| --- | --- | --- | --- | --- |
| **wild-type** | 15 | 13 | 2 | 87 |
|  | 10 | 10 | 0 | 100 |
|  | 16 | 15 | 1 | 94 |
| ***asna-1(ok938)*** | 10 | 2 | 8 | 20 |
|  | 15 | 4 | 11 | 27 |
|  | 12 | 5 | 7 | 42 |
| ***asna-1(deltaHis164)*** | 15 | 15 | 0 | 100 |
|  | 12 | 12 | 0 | 100 |
|  | 11 | 11 | 0 | 100 |

| **Statistical analysis:** |  |  |  |  |  |  |  |  |
| --- | --- | --- | --- | --- | --- | --- | --- | --- |
|  |  |  |  |  |  |  |  |  |
| **ANOVA summary** |  |  |  |  |  |  |  |  |
| F | 80,78 |  |  |  |  |  |  |  |
| P value | <,001 |  |  |  |  |  |  |  |
| P value summary | *** |  |  |  |  |  |  |  |
| Significant diff. among means (P < 0.05)? | Yes |  |  |  |  |  |  |  |
| R squared | 0,9642 |  |  |  |  |  |  |  |
|  |  |  |  |  |  |  |  |  |
| **ANOVA table** | SS | DF | MS | F (DFn, DFd) | P value |  |  |  |
| Treatment (between columns) | 9083 | 2 | 4541 | F (2, 6) = 80,78 | P<,001 |  |  |  |
| Residual (within columns) | 337,3 | 6 | 56,22 |  |  |  |  |  |
| Total | 9420 | 8 |  |  |  |  |  |  |
|  |  |  |  |  |  |  |  |  |
| **Normality of Residuals** |  |  |  |  |  |  |  |  |
| Test name | Statistics | P value | Passed normality test (alpha=0,05)? | P value summary | |  |  |  |
| D'Agostino-Pearson omnibus (K2) | 1,098 | 0,578 | Yes | ns |  |  |  |  |
| Anderson-Darling (A2*) | 0,3954 | 0,293 | Yes | ns |  |  |  |  |
| Shapiro-Wilk (W) | 0,9351 | 0,532 | Yes | ns |  |  |  |  |
| Kolmogorov-Smirnov (distance) | 0,2573 | 0,088 | Yes | ns |  |  |  |  |
|  |  |  |  |  |  |  |  |  |
| Number of families | 1 |  |  |  |  |  |  |  |
| Number of comparisons per family | 2 |  |  |  |  |  |  |  |
| Alpha | 0,05 |  |  |  |  |  |  |  |
|  |  |  |  |  |  |  |  |  |
| **Bonferroni's multiple comparisons test** | Mean Diff, | 95,00% CI of diff, | Below threshold? | Summary | Adjusted P Value | A-? |  |  |
| wild-type vs. asna-1(ok938) | 64 | 45,83 to 82,17 | Yes | *** | <,001 | B | asna-1(ok938) | |
| wild-type vs. asna-1(deltaHis164) | -6,333 | -24,51 to 11,84 | No | ns | 0,682 | C | asna-1(His164) | |
|  |  |  |  |  |  |  |  |  |
| **Test details** | Mean 1 | Mean 2 | Mean Diff, | SE of diff, | n1 | n2 | t | DF |
| wild-type vs. asna-1(ok938) | 93,67 | 29,67 | 64 | 6,122 | 3 | 3 | 10,45 | 6 |
| wild-type vs. asna-1(His164) | 93,67 | 100 | -6,333 | 6,122 | 3 | 3 | 1,034 | 6 |

**Fig 4B.** Comparison of germline and insulin/IIS defects in *asna-1(deltaHis164)* and *asna-1(ok938)* deletion mutants. Percentage of 1-day old adults with secreted DAF-28::GFP in coelomocytes. Experiments were performed in triplicate. Bars represent mean ± SD

|  | **total number of animals** | **no of animals with secreted DAF-28::GFP in coelomocytes** | **% of animals with cytoplasmic DAF-16::GFP** |
| --- | --- | --- | --- |
| **wild-type** | 12 | 12 | 100 |
|  | 23 | 23 | 100 |
|  | 14 | 14 | 100 |
| ***asna-1(ok938)*** | 12 | 0 | 0 |
|  | 10 | 0 | 0 |
|  | 13 | 0 | 0 |
| ***asna-1(deltaHis164)*** | 17 | 17 | 100 |
|  | 12 | 12 | 100 |
|  | 10 | 10 | 100 |

**Fig 5A**. Analysis of ASNA-1 point mutants reveals the importance of a conserved alanine for cisplatin resistance. Analysis of cisplatin sensitivity phenotype of seven million mutation project strains bearing point mutations in *asna-1*. Bars represent mean survival ± SD of 1-day-old adult animals exposed to 300 mg/mL of cisplatin for 24h.Statistical significance was determined by the one-way ANOVA followed by Bonferroni post-hoc correction. Survival experiments were performed in triplicate.

|  | **wild-type** | **VC40357 (gk592672)** | **VC20172 (gk181015)** | **VC40121 (gk472686)** | **VC40131 (gk4771176)** | **VC40531 (gk680952)** | **VC40833 (gk838146)** | **VC40546 (gk687101)** |
| --- | --- | --- | --- | --- | --- | --- | --- | --- |
| **% of survivial** | 91 | 12 | 93 | 93 | 95 | 74 | 98 | 50 |
|  | 93 | 3 | 96 | 91 | 83 | 80 | 88 | 62 |
|  | 94 | 1 | 69 | 85 | 92 | 82 | 92 | 56 |

| **Statistical analysis:** |  |  |  |  |  |  |  |  |
| --- | --- | --- | --- | --- | --- | --- | --- | --- |
|  |  |  |  |  |  |  |  |  |
| **ANOVA summary** |  |  |  |  |  |  |  |  |
| F | 56,19 |  |  |  |  |  |  |  |
| P value | <,001 |  |  |  |  |  |  |  |
| P value summary | *** |  |  |  |  |  |  |  |
| Significant diff. among means (P < 0.05)? | Yes |  |  |  |  |  |  |  |
| R squared | 0,9609 |  |  |  |  |  |  |  |
|  |  |  |  |  |  |  |  |  |
| **ANOVA table** | SS | DF | MS | F (DFn, DFd) | P value |  |  |  |
| Treatment (between columns) | 19209 | 7 | 2744 | F (7, 16) = 56,19 | P<,001 |  |  |  |
| Residual (within columns) | 781,3 | 16 | 48,83 |  |  |  |  |  |
| Total | 19991 | 23 |  |  |  |  |  |  |
|  |  |  |  |  |  |  |  |  |
| **Normality of Residuals** |  |  |  |  |  |  |  |  |
| Test name | Statistics | P value | Passed normality test (alpha=0,05)? | P value summary |  |  |  |  |
| D'Agostino-Pearson omnibus (K2) | 6,218 | 0,045 | No | * |  |  |  |  |
| Anderson-Darling (A2*) | 0,332 | 0,489 | Yes | ns |  |  |  |  |
| Shapiro-Wilk (W) | 0,9475 | 0,239 | Yes | ns |  |  |  |  |
| Kolmogorov-Smirnov (distance) | 0,09047 | 0,1 | Yes | ns |  |  |  |  |
|  |  |  |  |  |  |  |  |  |
| Number of families | 1 |  |  |  |  |  |  |  |
| Number of comparisons per family | 7 |  |  |  |  |  |  |  |
| Alpha | 0,05 |  |  |  |  |  |  |  |
|  |  |  |  |  |  |  |  |  |
| **Bonferroni's multiple comparisons test** | Mean Diff, | 95,00% CI of diff, | Below threshold? | Summary | Adjusted P Value | A-? |  |  |
| wild type vs. VC40357 (gk592672) | 87,33 | 69,75 to 104,9 | Yes | *** | <,001 | B | VC40357 (gk592672) |  |
| wild type vs. VC20172 (gk181015) | 6,667 | -10,92 to 24,25 | No | ns | >,999 | C | VC20172 (gk181015) |  |
| wild type vs. VC40121 (gk472686) | 3 | -14,59 to 20,59 | No | ns | >,999 | D | VC40121 (gk472686) |  |
| wild type vs. VC40131 (gk4771176) | 2,667 | -14,92 to 20,25 | No | ns | >,999 | E | VC40131 (gk4771176) |  |
| wild type vs. VC40531 (gk680952) | 14 | -3,586 to 31,59 | No | ns | 0,182 | F | VC40531 (gk680952) |  |
| wild type vs. VC40833 (gk838146) | 0 | -17,59 to 17,59 | No | ns | >,999 | G | VC40833 (gk838146) |  |
| wild type vs. VC40546 (gk687101) | 36,67 | 19,08 to 54,25 | Yes | *** | <,001 | H | VC40546 (gk687101) |  |
|  |  |  |  |  |  |  |  |  |
| **Test details** | Mean 1 | Mean 2 | Mean Diff, | SE of diff, | n1 | n2 | t | DF |
| wild type vs. VC40357 (gk592672) | 92,67 | 5,333 | 87,33 | 5,706 | 3 | 3 | 15,31 | 16 |
| wild type vs. VC20172 (gk181015) | 92,67 | 86 | 6,667 | 5,706 | 3 | 3 | 1,168 | 16 |
| wild type vs. VC40121 (gk472686) | 92,67 | 89,67 | 3 | 5,706 | 3 | 3 | 0,5258 | 16 |
| wild type vs. VC40131 (gk4771176) | 92,67 | 90 | 2,667 | 5,706 | 3 | 3 | 0,4674 | 16 |
| wild type vs. VC40531 (gk680952) | 92,67 | 78,67 | 14 | 5,706 | 3 | 3 | 2,454 | 16 |
| wild type vs. VC40833 (gk838146) | 92,67 | 92,67 | 0 | 5,706 | 3 | 3 | 0 | 16 |
| wild type vs. VC40546 (gk687101) | 92,67 | 56 | 36,67 | 5,706 | 3 | 3 | 6,426 | 16 |

**Fig 5B.** Analysis of ASNA-1 point mutants reveals the importance of a conserved alanine for cisplatin resistance. Rescue of the cisplatin sensitivity phenotype of *asna-1(ok938)* deletion mutants and *asna-1(gk592672 /A63V)* mutants with a single copy ASNA-1(+) transgene *knuSi184.*

Bars represent mean survival ± SD of 1-day-old adult animals exposed to 300 mg/mL of cisplatin for 24h. Statistical significance was determined by the one-way ANOVA followed by Bonferroni post-hoc correction. Survival experiments were performed in triplicate.

| **% of survivial** | **wild-type** | ***asna-1(ok938)*** | ***asna-1(ok938);knuSi184*** | ***asna-1(A63V)*** | ***asna-1(A63V);knuSi184*** |
| --- | --- | --- | --- | --- | --- |
|  | 99 | 53 | 93 | 55 | 95 |
|  | 94 | 51 | 83 | 53 | 91 |
|  | 89 | 56 | 87 | 56 | 95 |
|  | 93 | 34 | 56 |  |  |
|  | 93 | 54 | 81 |  |  |

| **Statistical analysis:** |  |  |  |  |  |  |  |  |
| --- | --- | --- | --- | --- | --- | --- | --- | --- |
|  |  |  |  |  |  |  |  |  |
| **ANOVA summary** |  |  |  |  |  |  |  |  |
| F | 24,78 |  |  |  |  |  |  |  |
| P value | <,001 |  |  |  |  |  |  |  |
| P value summary | *** |  |  |  |  |  |  |  |
| Significant diff. among means (P < 0.05)? | Yes |  |  |  |  |  |  |  |
| R squared | 0,861 |  |  |  |  |  |  |  |
|  |  |  |  |  |  |  |  |  |
| **ANOVA table** | SS | DF | MS | F (DFn, DFd) | P value |  |  |  |
| Treatment (between columns) | 7357 | 4 | 1839 | F (4, 16) = 24,78 | P<,001 |  |  |  |
| Residual (within columns) | 1188 | 16 | 74,23 |  |  |  |  |  |
| Total | 8545 | 20 |  |  |  |  |  |  |
|  |  |  |  |  |  |  |  |  |
| **Normality of Residuals** |  |  |  |  |  |  |  |  |
| Test name | Statistics | P value | Passed normality test (alpha=0,05)? | P value summary |  |  |  |  |
| D'Agostino-Pearson omnibus (K2) | 17,27 | <,001 | No | *** |  |  |  |  |
| Anderson-Darling (A2*) | 1,521 | <,001 | No | *** |  |  |  |  |
| Shapiro-Wilk (W) | 0,8157 | 0,001 | No | ** |  |  |  |  |
| Kolmogorov-Smirnov (distance) | 0,2309 | 0,005 | No | ** |  |  |  |  |
|  |  |  |  |  |  |  |  |  |
| Number of families | 1 |  |  |  |  |  |  |  |
| Number of comparisons per family | 5 |  |  |  |  |  |  |  |
| Alpha | 0,05 |  |  |  |  |  |  |  |
|  |  |  |  |  |  |  |  |  |
| **Bonferroni's multiple comparisons test** | Mean Diff, | 95,00% CI of diff, | Below threshold? | Summary | Adjusted P Value |  |  |  |
| wild-type vs. asna-1(ok938) | 44 | 28,08 to 59,92 | Yes | *** | <,001 | A-B |  |  |
| wild-type vs. asna-1(ok938);knuSi184 | 13,6 | -2,316 to 29,52 | No | ns | 0,119 | A-C |  |  |
| wild-type vs. asna-1(A63V) | 38,93 | 20,56 to 57,31 | Yes | *** | <,001 | A-D |  |  |
| wild-type vs. asna-1(A63V);knuSi184 | -0,06667 | -18,44 to 18,31 | No | ns | >,999 | A-E |  |  |
| asna-1(ok938) vs. asna-1(A63V) | -5,067 | -23,44 to 13,31 | No | ns | >,999 | B-D |  |  |
|  |  |  |  |  |  |  |  |  |
| **Test details** | Mean 1 | Mean 2 | Mean Diff, | SE of diff, | n1 | n2 | t | DF |
| wild-type vs. asna-1(ok938) | 93,6 | 49,6 | 44 | 5,449 | 5 | 5 | 8,075 | 16 |
| wild-type vs. asna-1(ok938);knuSi184 | 93,6 | 80 | 13,6 | 5,449 | 5 | 5 | 2,496 | 16 |
| wild-type vs. asna-1(A63V) | 93,6 | 54,67 | 38,93 | 6,292 | 5 | 3 | 6,188 | 16 |
| wild-type vs. asna-1(A63V);knuSi184 | 93,6 | 93,67 | -0,06667 | 6,292 | 5 | 3 | 0,0106 | 16 |
| asna-1(ok938) vs. asna-1(A63V) | 49,6 | 54,67 | -5,067 | 6,292 | 5 | 3 | 0,8052 | 16 |

**Fig 5D**. Analysis of ASNA-1 point mutants reveals the importance of a conserved alanine for cisplatin resistance. Brood size analysis of wild-type and *asna-1(A63V)* mutants (n=5). Statistical significance was determined by the independent two-sample t-test. Bars represent mean ± SD.

| **wild-type** | ***asna-1(A63V)*** |
| --- | --- |
| 240 | 173 |
| 197 | 244 |
| 211 | 227 |
| 204 | 192 |
| 214 | 198 |

| **Statistical analysis:** |  |
| --- | --- |
|  |  |
|  |  |
| **Table Analyzed** |  |
| Column B | asna-1(A63V) |
| vs. | vs, |
| Column A | wild-type |
|  |  |
| **Unpaired t test** |  |
| P value | 0,6741 |
| P value summary | ns |
| Significantly different (P < 0.05)? | No |
| One- or two-tailed P value? | Two-tailed |
| t, df | t=0,4364, df=8 |
|  |  |
| **How big is the difference?** |  |
| Mean of column A | 213,2 |
| Mean of column B | 206,8 |
| Difference between means (B - A) ± SEM | -6,400 ± 14,67 |
| 95% confidence interval | -40,22 to 27,42 |
| R squared (eta squared) | 0,02325 |
|  |  |
| **F test to compare variances** |  |
| F, DFn, Dfd | 3,017, 4, 4 |
| P value | 0,3101 |
| P value summary | ns |
| Significantly different (P < 0.05)? | No |
|  |  |
| **Data analyzed** |  |
| Sample size, column A | 5 |
| Sample size, column B | 5 |

**Fig 5E**. Analysis of ASNA-1 point mutants reveals the importance of a conserved alanine for cisplatin resistance. Life span analysis of wild-type (n=40) and *asna-1(A63V)* (n=40) animals.

|  | **Time (days)** | **wild-type** | ***asna-1(A63V)*** |
| --- | --- | --- | --- |
| **% alive** | 0 | 100 | 100 |
|  | 4 | 98 | 100 |
|  | 5 | 95 | 100 |
|  | 8 | 85 | 98 |
|  | 10 | 83 | 87 |
|  | 11 | 34 | 53 |
|  | 14 | 11 | 28 |
|  | 15 | 0 | 17 |
|  | 16 | 0 | 7 |
|  | 17 | 0 | 0 |

**Fig 5G**. Analysis of ASNA-1 point mutants reveals the importance of a conserved alanine for cisplatin resistance. Percentage of 1-day old adults with indicated genotypes displaying only cytoplasmic localized DAF-16::GFP. Experiments were performed in triplicate. Statistical significance was determined by the one-way ANOVA followed by Bonferroni post-hoc correction. Bars represent mean ± SD.

|  | **total number of animals** | **no of animals with cytoplasmic DAF-16::GFP** | **no of animals with nuclear DAF-16::GFP** | **% of animals with cytoplasmic DAF-16::GFP** |
| --- | --- | --- | --- | --- |
| **wild-type** | 15 | 13 | 2 | 87 |
|  | 10 | 10 | 0 | 100 |
|  | 16 | 15 | 1 | 94 |
| ***asna-1(ok938)*** | 10 | 2 | 8 | 20 |
|  | 15 | 4 | 11 | 27 |
|  | 12 | 5 | 7 | 42 |
| ***asna-1(A63V)*** | 10 | 10 | 0 | 100 |
|  | 12 | 12 | 0 | 100 |
|  | 9 | 9 | 0 | 100 |

| **Statistical analysis:** |  |  |  |  |  |  |  |  |
| --- | --- | --- | --- | --- | --- | --- | --- | --- |
|  |  |  |  |  |  |  |  |  |
|  |  |  |  |  |  |  |  |  |
| **ANOVA summary** |  |  |  |  |  |  |  |  |
| F | 80,78 |  |  |  |  |  |  |  |
| P value | <,001 |  |  |  |  |  |  |  |
| P value summary | *** |  |  |  |  |  |  |  |
| Significant diff. among means (P < 0.05)? | Yes |  |  |  |  |  |  |  |
| R squared | 0,9642 |  |  |  |  |  |  |  |
|  |  |  |  |  |  |  |  |  |
| **ANOVA table** | SS | DF | MS | F (DFn, DFd) | P value |  |  |  |
| Treatment (between columns) | 9083 | 2 | 4541 | F (2, 6) = 80,78 | P<,001 |  |  |  |
| Residual (within columns) | 337,3 | 6 | 56,22 |  |  |  |  |  |
| Total | 9420 | 8 |  |  |  |  |  |  |
|  |  |  |  |  |  |  |  |  |
| **Normality of Residuals** |  |  |  |  |  |  |  |  |
| Test name | Statistics | P value | Passed normality test (alpha=0,05)? | P value summary |  |  |  |  |
| D'Agostino-Pearson omnibus (K2) | 1,098 | 0,578 | Yes | ns |  |  |  |  |
| Anderson-Darling (A2*) | 0,3954 | 0,293 | Yes | ns |  |  |  |  |
| Shapiro-Wilk (W) | 0,9351 | 0,532 | Yes | ns |  |  |  |  |
| Kolmogorov-Smirnov (distance) | 0,2573 | 0,088 | Yes | ns |  |  |  |  |
|  |  |  |  |  |  |  |  |  |
| Number of families | 1 |  |  |  |  |  |  |  |
| Number of comparisons per family | 2 |  |  |  |  |  |  |  |
| Alpha | 0,05 |  |  |  |  |  |  |  |
|  |  |  |  |  |  |  |  |  |
| **Bonferroni's multiple comparisons test** | Mean Diff, | 95,00% CI of diff, | Below threshold? | Summary | Adjusted P Value | A-? |  |  |
| wild-type vs. asna-1(ok938) | 64 | 45,83 to 82,17 | Yes | *** | <,001 | B | asna-1(ok938) | |
| wild-type vs. asna-1(A63V) | -6,333 | -24,51 to 11,84 | No | ns | 0,682 | C | asna-1(A63V) |  |
|  |  |  |  |  |  |  |  |  |
| **Test details** | Mean 1 | Mean 2 | Mean Diff, | SE of diff, | n1 | n2 | t | DF |
| wild-type vs. asna-1(ok938) | 93,67 | 29,67 | 64 | 6,122 | 3 | 3 | 10,45 | 6 |
| wild-type vs. asna-1(A63V) | 93,67 | 100 | -6,333 | 6,122 | 3 | 3 | 1,034 | 6 |

**Fig 5H.** Analysis of ASNA-1 point mutants reveals the importance of a conserved alanine for cisplatin resistance. Percentage of 1-day old adults with secreted DAF-28::GFP in coelomocytes. Experiments were performed in triplicate. Bars represent mean ± SD.

|  | **total number of animals** | **no of animals with secreted DAF-28::GFP in coelomocytes** | **% of animals with cytoplasmic DAF-16::GFP** |
| --- | --- | --- | --- |
| **wild-type** | 12 | 12 | 100 |
|  | 23 | 23 | 100 |
|  | 14 | 14 | 100 |
| ***asna-1(ok938)*** | 12 | 0 | 0 |
|  | 10 | 0 | 0 |
|  | 13 | 0 | 0 |
| ***asna-1(A63V)*** | 9 | 9 | 100 |
|  | 30 | 30 | 100 |
|  | 14 | 14 | 100 |

**Fig 6B***. asna-1(A63V)* mutants are defective for TAP insertion but have normal insulin secretion.

Pearson’s correlation analysis of GFP::SEC-61β and mCherry::SP12 co-localization in different strains. The box plot represents the average Pearson correlation coefficient (R) of the indicated strains.

Statistical significance was determined by the Mann-Whitney test (n ≥ 10).

|  | ***asna-1(+)*** | ***asna-1(A63V)*** |
| --- | --- | --- |
| Pearson correlation coefficient (R) | 0,878 | 0,868 |
|  | 0,894 | 0,827 |
|  | 0,803 | 0,811 |
|  | 0,93 | 0,902 |
|  | 0,718 | 0,869 |
|  | 0,817 | 0,853 |
|  | 0,913 | 0,827 |
|  | 0,95 | 0,894 |
|  | 0,96 | 0,873 |
|  | 0,896 | 0,652 |
|  | 0,972 | 0,848 |
|  | 0,926 | 0,867 |
|  | 0,905 | 0,861 |
|  |  | 0,829 |
|  |  | 0,856 |
|  |  | 0,883 |
|  |  | 0,917 |
|  |  | 0,859 |
|  |  | 0,812 |

| **Statistical analysis:** |  |
| --- | --- |
|  |  |
| Column B | asna-1(A63V) |
| vs. | vs, |
| Column A | asna-1(+) |
|  |  |
| **Mann Whitney test** |  |
| P value | 0,018 |
| Exact or approximate P value? | Exact |
| P value summary | * |
| Significantly different (P < 0.05)? | Yes |
| One- or two-tailed P value? | Two-tailed |
| Sum of ranks in column A,B | 275,5 , 252,5 |
| Mann-Whitney U | 62,5 |
|  |  |
| **Difference between medians** |  |
| Median of column A | 0,9050, n=13 |
| Median of column B | 0,8590, n=19 |
| Difference: Actual | -0,046 |
| Difference: Hodges-Lehmann | -0,049 |

**Fig 6C**. *asna-1(A63V)* mutants are defective for TAP insertion but have normal insulin secretion.

Band intensity quantification of membrane/cytosolic fraction of ASNA-1::GFP and ASNA1^A63V^::GFP based on the western blots presented in Fig S11. Statistical significance was determined by the independent two-sample t-test. Error bars represent ± SD.

|  | **ASNA-1::GFP** | **ASNA-1(A63V)::GFP** |
| --- | --- | --- |
| Membranes fraction/cytoplasmic fraction | 13,66171373 | 8,91957052 |
|  | 11,63973138 | 6,207680497 |
|  | 19,79499863 | 10,33069827 |

| **Statistical analysis:** |  |  |  |  |
| --- | --- | --- | --- | --- |
|  |  |  |  |  |
| Column B | ASNA-1(A63V)::GFP |  |  |  |
| vs. | vs, |  |  |  |
| Column A | ASNA-1::GFP |  |  |  |
|  |  |  |  |  |
| **Paired t test** |  |  |  |  |
| P value | 0,047 |  |  |  |
| P value summary | * |  |  |  |
| Significantly different (P < 0.05)? | Yes |  |  |  |
| One- or two-tailed P value? | Two-tailed |  |  |  |
| t, df | t=4,445, df=2 |  |  |  |
| Number of pairs | 3 |  |  |  |
|  |  |  |  |  |
| **How big is the difference?** |  |  |  |  |
| Mean of differences (B - A) | -6,546 |  |  |  |
| SD of differences | 2,551 |  |  |  |
| SEM of differences | 1,473 |  |  |  |
| 95% confidence interval | -12,88 to -0,2101 |  |  |  |
| R squared (partial eta squared) | 0,9081 |  |  |  |
|  |  |  |  |  |
| **How effective was the pairing?** |  |  |  |  |
| Correlation coefficient (r) | 0,8945 |  |  |  |
| P value (one tailed) | 0,148 |  |  |  |
| P value summary | ns |  |  |  |
| Was the pairing significantly effective? | No |  |  |  |
|  |  |  |  |  |
| **Normality of Residuals** |  |  |  |  |
| Test name | Statistics | P value | Passed normality test (alpha=0,05)? | P value summary |
| Anderson-Darling (A2*) | N too small |  |  |  |
| D'Agostino-Pearson omnibus (K2) | N too small |  |  |  |
| Shapiro-Wilk (W) | 0,8569 | 0,259 | Yes | ns |
| Kolmogorov-Smirnov (distance) | N too small |  |  |  |

**Fig 6D.** *asna-1(A63V)* mutants are defective for TAP insertion but have normal insulin secretion.

Graph represents the band intensity quantification of oxidized/reduced ASNA-1^A63V^::GFP. Statistical significance was determined by the independent two-sample t-test. Experiments were performed in triplicate. Bars represent ± SD.

|  | **ASNA-1::GFP** | **ASNA-1(A63V)::GFP** |
| --- | --- | --- |
| Oxidized/reduced ASNA-1(A63V) (Relative to ASNA-1::GFP) (%) | 100 | 144,106212 |
|  | 100 | 166,07515 |
|  | 100 | 195,63643 |
|  | 100 | 184,04334 |

| **Statistical analysis:** |  |  |  |  |
| --- | --- | --- | --- | --- |
|  |  |  |  |  |
| Column B | ASNA-1(A63V)::GFP |  |  |  |
| vs. | vs, |  |  |  |
| Column A | ASNA-1::GFP |  |  |  |
|  |  |  |  |  |
| **Unpaired t test** |  |  |  |  |
| P value | <,001 |  |  |  |
| P value summary | *** |  |  |  |
| Significantly different (P < 0.05)? | Yes |  |  |  |
| One- or two-tailed P value? | Two-tailed |  |  |  |
| t, df | t=6,447, df=6 |  |  |  |
|  |  |  |  |  |
| **How big is the difference?** |  |  |  |  |
| Mean of column A | 100 |  |  |  |
| Mean of column B | 172,5 |  |  |  |
| Difference between means (B - A) ± SEM | 72,47 ± 11,24 |  |  |  |
| 95% confidence interval | 44,96 to 99,97 |  |  |  |
| R squared (eta squared) | 0,8739 |  |  |  |
|  |  |  |  |  |
| **F test to compare variances** |  |  |  |  |
| F, DFn, Dfd | Infinity, 3, 3 |  |  |  |
| P value | <,001 |  |  |  |
| P value summary | *** |  |  |  |
| Significantly different (P < 0.05)? | Yes |  |  |  |
|  |  |  |  |  |
| **Normality of Residuals** |  |  |  |  |
| Test name | Statistics | P value | Passed normality test (alpha=0,05)? | P value summary |
| Anderson-Darling (A2*) | 0,5552 | 0,103 | Yes | ns |
| D'Agostino-Pearson omnibus (K2) | 2,371 | 0,306 | Yes | ns |
| Shapiro-Wilk (W) | 0,8963 | 0,268 | Yes | ns |
| Kolmogorov-Smirnov (distance) | 0,25 | 0,1 | Yes | ns |
|  |  |  |  |  |
| **Data analyzed** |  |  |  |  |
| Sample size, column A | 4 |  |  |  |
| Sample size, column B | 4 |  |  |  |

**Fig 6E**. *asna-1(A63V)* mutants are defective for TAP insertion but have normal insulin secretion.

Graph represents the band intensity quantification of oxidized/reduced ASNA-1^A63V^::GFP. Statistical significance was determined by the independent two-sample t-test. Experiments were performed in triplicate. Bars represent ± SD.

|  | **ASNA-1(A63V)::GFP** | **ASNA-1(A63V)::GFP +CP** |
| --- | --- | --- |
| Oxidized/reduced ASNA-1(A63V)::GFP+CP (Relative to ASNA-1(A63V)::GFP) (%) | 100 | 132,1941765 |
|  |  |  |
|  | 100 | 114,7723378 |
|  |  |  |

| **Statistical analysis:** |  |  |  |  |
| --- | --- | --- | --- | --- |
|  |  |  |  |  |
| Column B | ASNA-1(A63V)::GFP +CP | |  |  |
| vs. | vs, |  |  |  |
| Column A | ASNA-1(A63V)::GFP |  |  |  |
|  |  |  |  |  |
| **Unpaired t test** |  |  |  |  |
| P value | 0,114 |  |  |  |
| P value summary | ns |  |  |  |
| Significantly different (P < 0.05)? | No |  |  |  |
| One- or two-tailed P value? | Two-tailed |  |  |  |
| t, df | t=2,696, df=2 |  |  |  |
|  |  |  |  |  |
| **How big is the difference?** |  |  |  |  |
| Mean of column A | 100 |  |  |  |
| Mean of column B | 123,5 |  |  |  |
| Difference between means (B - A) ± SEM | 23,48 ± 8,711 |  |  |  |
| 95% confidence interval | -14,00 to 60,96 |  |  |  |
| R squared (eta squared) | 0,7842 |  |  |  |
|  |  |  |  |  |
| **F test to compare variances** |  |  |  |  |
| F, DFn, Dfd |  |  |  |  |
| P value |  |  |  |  |
| P value summary |  |  |  |  |
| Significantly different (P < 0.05)? |  |  |  |  |
|  |  |  |  |  |
| **Normality of Residuals** |  |  |  |  |
| Test name | Statistics | P value | Passed normality test (alpha=0,05)? | P value summary |
| Anderson-Darling (A2*) | N too small |  |  |  |
| D'Agostino-Pearson omnibus (K2) | N too small |  |  |  |
| Shapiro-Wilk (W) | 0,9447 | 0,683 | Yes | ns |
| Kolmogorov-Smirnov (distance) | N too small |  |  |  |
|  |  |  |  |  |
| **Data analyzed** |  |  |  |  |
| Sample size, column A | 2 |  |  |  |
| Sample size, column B | 2 |  |  |  |

**S7C Fig**. *hsp-4p::GFP* expression quantification in the wild-type and *asna-1(A63V)* animals (n=5).

|  | **wild-type** | ***asna-1(A63V)*** |
| --- | --- | --- |
| hsp-4:GFP expression quantification | 123,177864 | 128,437878 |
|  | 103,035449 | 54,9484103 |
|  | 77,3821018 | 76,5867437 |
|  | 106,769657 | 172,989134 |
|  | 89,6349282 | 67,2040418 |

| **Statistical analysis:** |  |  |  |  |
| --- | --- | --- | --- | --- |
|  |  |  |  |  |
| Column B | asna-1(A63V) |  |  |  |
| vs. | vs, |  |  |  |
| Column A | wild-type |  |  |  |
|  |  |  |  |  |
| **Unpaired t test** |  |  |  |  |
| P value | 0,999 |  |  |  |
| P value summary | ns |  |  |  |
| Significantly different (P < 0.05)? | No |  |  |  |
| One- or two-tailed P value? | Two-tailed |  |  |  |
| t, df | t=0,001417, df=8 |  |  |  |
|  |  |  |  |  |
| **How big is the difference?** |  |  |  |  |
| Mean of column A | 100 |  |  |  |
| Mean of column B | 100 |  |  |  |
| Difference between means (B - A) ± SEM | 0,03324 ± 23,45 |  |  |  |
| 95% confidence interval | -54,05 to 54,12 |  |  |  |
| R squared (eta squared) | 2,511E-07 |  |  |  |
|  |  |  |  |  |
| **F test to compare variances** |  |  |  |  |
| F, DFn, Dfd | 8,082, 4, 4 |  |  |  |
| P value | 0,067 |  |  |  |
| P value summary | ns |  |  |  |
| Significantly different (P < 0.05)? | No |  |  |  |
|  |  |  |  |  |
| **Normality of Residuals** |  |  |  |  |
| Test name | Statistics | P value | Passed normality test (alpha=0,05)? | P value summary |
| Anderson-Darling (A2*) | 0,2549 | 0,645 | Yes | ns |
| D'Agostino-Pearson omnibus (K2) | 2,286 | 0,319 | Yes | ns |
| Shapiro-Wilk (W) | 0,9459 | 0,62 | Yes | ns |
| Kolmogorov-Smirnov (distance) | 0,1412 | 0,1 | Yes | ns |
|  |  |  |  |  |
| **Data analyzed** |  |  |  |  |
| Sample size, column A | 5 |  |  |  |
| Sample size, column B | 5 |  |  |  |

**S7D Fig.** Relative mRNA analysis of ER stress reporter *hsp-4* in 1-day old adult *asna-1(A63V)* animals. Statistical significance was determined by the independent two-sample t-test.

Experiments were performed in triplicates. F44B9.5 was used as a normalizing control. Bars represent mean ± SEM.

|  | **wild-type** | ***asna-1(A63V)*** |
| --- | --- | --- |
| Relative gene expression | 0,147 | 0,558 |
|  | 0,049 | -0,14 |
|  | -0,2 | 0,235 |

| **Statistical analysis:** |  |  |  |  |
| --- | --- | --- | --- | --- |
|  |  |  |  |  |
| Column B | asna-1(A63V) |  |  |  |
| vs. | vs, |  |  |  |
| Column A | wild-type |  |  |  |
|  |  |  |  |  |
| **Unpaired t test** |  |  |  |  |
| P value | 0,389 |  |  |  |
| P value summary | ns |  |  |  |
| Significantly different (P < 0.05)? | No |  |  |  |
| One- or two-tailed P value? | Two-tailed |  |  |  |
| t, df | t=0,9665, df=4 |  |  |  |
|  |  |  |  |  |
| **How big is the difference?** |  |  |  |  |
| Mean of column A | -0,001333 |  |  |  |
| Mean of column B | 0,2177 |  |  |  |
| Difference between means (B - A) ± SEM | 0,2190 ± 0,2266 |  |  |  |
| 95% confidence interval | -0,4101 to 0,8481 |  |  |  |
| R squared (eta squared) | 0,1893 |  |  |  |
|  |  |  |  |  |
| **F test to compare variances** |  |  |  |  |
| F, DFn, Dfd | 3,813, 2, 2 |  |  |  |
| P value | 0,416 |  |  |  |
| P value summary | ns |  |  |  |
| Significantly different (P < 0.05)? | No |  |  |  |
|  |  |  |  |  |
| **Normality of Residuals** |  |  |  |  |
| Test name | Statistics | P value | Passed normality test (alpha=0,05)? | P value summary |
| Anderson-Darling (A2*) | N too small |  |  |  |
| D'Agostino-Pearson omnibus (K2) | N too small |  |  |  |
| Shapiro-Wilk (W) | 0,9788 | 0,945 | Yes | ns |
| Kolmogorov-Smirnov (distance) | 0,1945 | 0,1 | Yes | ns |
|  |  |  |  |  |
| **Data analyzed** |  |  |  |  |
| Sample size, column A | 3 |  |  |  |
| Sample size, column B | 3 |  |  |  |

**S7E Fig**. Relative mRNA analysis of mitochondrial stress reporters (*hsp-6* and *hsp-60*) in 1-day old adult *asna-1(A63V)* animals. Statistical significance was determined by the independent two-sample t-test. Experiments were performed in triplicates. F44B9.5 was used as a normalizing control. Bars represent mean ± SEM.

|  | **wild-type** | | | ***asna-1(A63V)*** | | |
| --- | --- | --- | --- | --- | --- | --- |
| ***hsp-6*** | 0,149 | 0,094 | -0,24 | 0,586 | -0,01 | 0,302 |
| ***hsp-60*** | 0,033 | -0,06 | 0,026 | 0,113 | -0,19 | -0,1 |

| **Statistical analysis:** |  |  |  |  |
| --- | --- | --- | --- | --- |
|  |  |  |  |  |
| **Table Analyzed** | **hsp-6** |  |  |  |
| Column B | asna-1(A63V) |  |  |  |
| vs. | vs, |  |  |  |
| Column A | wild-type |  |  |  |
|  |  |  |  |  |
| **Unpaired t test** |  |  |  |  |
| P value | 0,238 |  |  |  |
| P value summary | ns |  |  |  |
| Significantly different (P < 0.05)? | No |  |  |  |
| One- or two-tailed P value? | Two-tailed |  |  |  |
| t, df | t=1,384, df=4 |  |  |  |
|  |  |  |  |  |
| **How big is the difference?** |  |  |  |  |
| Mean of column A | 0,001 |  |  |  |
| Mean of column B | 0,2927 |  |  |  |
| Difference between means (B - A) ± SEM | 0,2917 ± 0,2107 |  |  |  |
| 95% confidence interval | -0,2933 to 0,8767 |  |  |  |
| R squared (eta squared) | 0,3239 |  |  |  |
|  |  |  |  |  |
| **F test to compare variances** |  |  |  |  |
| F, DFn, Dfd | 2,005, 2, 2 |  |  |  |
| P value | 0,665 |  |  |  |
| P value summary | ns |  |  |  |
| Significantly different (P < 0.05)? | No |  |  |  |
|  |  |  |  |  |
| **Normality of Residuals** |  |  |  |  |
| Test name | Statistics | P value | Passed normality test (alpha=0,05)? | P value summary |
| Anderson-Darling (A2*) | N too small |  |  |  |
| D'Agostino-Pearson omnibus (K2) | N too small |  |  |  |
| Shapiro-Wilk (W) | 0,9378 | 0,641 | Yes | ns |
| Kolmogorov-Smirnov (distance) | 0,1851 | 0,1 | Yes | ns |
|  |  |  |  |  |
| Data analyzed |  |  |  |  |
| Sample size, column A | 3 |  |  |  |
| Sample size, column B | 3 |  |  |  |
|  |  |  |  |  |
|  |  |  |  |  |
|  |  |  |  |  |
| **Table Analyzed** | **hsp-60** |  |  |  |
| Column B | asna-1(A63V) |  |  |  |
| vs. | vs, |  |  |  |
| Column A | wild-type |  |  |  |
|  |  |  |  |  |
| **Unpaired t test** |  |  |  |  |
| P value | 0,569 |  |  |  |
| P value summary | ns |  |  |  |
| Significantly different (P < 0.05)? | No |  |  |  |
| One- or two-tailed P value? | Two-tailed |  |  |  |
| t, df | t=0,6196, df=4 |  |  |  |
|  |  |  |  |  |
| **How big is the difference?** |  |  |  |  |
| Mean of column A | -0,0003333 |  |  |  |
| Mean of column B | -0,059 |  |  |  |
| Difference between means (B - A) ± SEM | -0,05867 ± 0,09468 |  |  |  |
| 95% confidence interval | -0,3216 to 0,2042 |  |  |  |
| R squared (eta squared) | 0,08757 |  |  |  |
|  |  |  |  |  |
| **F test to compare variances** |  |  |  |  |
| F, DFn, Dfd | 9,027, 2, 2 |  |  |  |
| P value | 0,199 |  |  |  |
| P value summary | ns |  |  |  |
| Significantly different (P < 0.05)? | No |  |  |  |
|  |  |  |  |  |
| **Normality of Residuals** |  |  |  |  |
| Test name | Statistics | P value | Passed normality test (alpha=0,05)? | P value summary |
| Anderson-Darling (A2*) | N too small |  |  |  |
| D'Agostino-Pearson omnibus (K2) | N too small |  |  |  |
| Shapiro-Wilk (W) | 0,9568 | 0,795 | Yes | ns |
| Kolmogorov-Smirnov (distance) | 0,2073 | 0,1 | Yes | ns |
|  |  |  |  |  |
| Data analyzed |  |  |  |  |
| Sample size, column A | 3 |  |  |  |
| Sample size, column B | 3 |  |  |  |

**S7F Fig.** Relative mRNA analysis of oxidative stress reporters (*gst-4, gst-30*, and *gst-38*) in 1-day old adult *asna-1(A63V)* animals. Statistical significance was determined by the independent two-sample t-test. Experiments were performed in triplicates. F44B9.5 was used as a normalizing control. Bars represent mean ± SEM.

|  | **wild-type** | | | ***asna-1(A63V)*** | | |
| --- | --- | --- | --- | --- | --- | --- |
| gst-4 | -0,02 | 0,033 | -0,01 | -0,13 | -0,07 | -0,09 |
| gst-30 | -0,03 | 0,038 | -0,01 | 0,144 | 0,167 | 0,19 |
| gst-38 | -0,02 | -0,01 | 0,027 | 0,248 | 0,21 | 0,238 |

| **Statistical analysis:** |  |  |  |  |
| --- | --- | --- | --- | --- |
|  |  |  |  |  |
| **Table Analyzed** | **gst-4** |  |  |  |
| Column B | asna-1(A63V) |  |  |  |
| vs. | vs, |  |  |  |
| Column A | wild-type |  |  |  |
|  |  |  |  |  |
| **Unpaired t test** |  |  |  |  |
| P value | 0,015 |  |  |  |
| P value summary | * |  |  |  |
| Significantly different (P < 0.05)? | Yes |  |  |  |
| One- or two-tailed P value? | Two-tailed |  |  |  |
| t, df | t=4,071, df=4 |  |  |  |
|  |  |  |  |  |
| **How big is the difference?** |  |  |  |  |
| Mean of column A | 0,001 |  |  |  |
| Mean of column B | -0,09667 |  |  |  |
| Difference between means (B - A) ± SEM | -0,09767 ± 0,02399 |  |  |  |
| 95% confidence interval | -0,1643 to -0,03106 |  |  |  |
| R squared (eta squared) | 0,8056 |  |  |  |
|  |  |  |  |  |
| **F test to compare variances** |  |  |  |  |
| F, DFn, Dfd | 1,177, 2, 2 |  |  |  |
| P value | 0,919 |  |  |  |
| P value summary | ns |  |  |  |
| Significantly different (P < 0.05)? | No |  |  |  |
|  |  |  |  |  |
| **Normality of Residuals** |  |  |  |  |
| Test name | Statistics | P value | Passed normality test (alpha=0,05)? | P value summary |
| Anderson-Darling (A2*) | N too small |  |  |  |
| D'Agostino-Pearson omnibus (K2) | N too small |  |  |  |
| Shapiro-Wilk (W) | 0,9393 | 0,653 | Yes | ns |
| Kolmogorov-Smirnov (distance) | 0,1782 | 0,1 | Yes | ns |
|  |  |  |  |  |
| Data analyzed |  |  |  |  |
| Sample size, column A | 3 |  |  |  |
| Sample size, column B | 3 |  |  |  |
|  |  |  |  |  |
|  |  |  |  |  |
| **Table Analyzed** | **gst-30** |  |  |  |
| Column B | asna-1(A63V) |  |  |  |
| vs. | vs, |  |  |  |
| Column A | wild-type |  |  |  |
|  |  |  |  |  |
| **Unpaired t test** |  |  |  |  |
| P value | 0,002 |  |  |  |
| P value summary | ** |  |  |  |
| Significantly different (P < 0.05)? | Yes |  |  |  |
| One- or two-tailed P value? | Two-tailed |  |  |  |
| t, df | t=6,941, df=4 |  |  |  |
|  |  |  |  |  |
| **How big is the difference?** |  |  |  |  |
| Mean of column A | -0,0006667 |  |  |  |
| Mean of column B | 0,167 |  |  |  |
| Difference between means (B - A) ± SEM | 0,1677 ± 0,02415 |  |  |  |
| 95% confidence interval | 0,1006 to 0,2347 |  |  |  |
| R squared (eta squared) | 0,9233 |  |  |  |
|  |  |  |  |  |
| **F test to compare variances** |  |  |  |  |
| F, DFn, Dfd | 2,309, 2, 2 |  |  |  |
| P value | 0,604 |  |  |  |
| P value summary | ns |  |  |  |
| Significantly different (P < 0.05)? | No |  |  |  |
|  |  |  |  |  |
| **Normality of Residuals** |  |  |  |  |
| Test name | Statistics | P value | Passed normality test (alpha=0,05)? | P value summary |
| Anderson-Darling (A2*) | N too small |  |  |  |
| D'Agostino-Pearson omnibus (K2) | N too small |  |  |  |
| Shapiro-Wilk (W) | 0,9431 | 0,684 | Yes | ns |
| Kolmogorov-Smirnov (distance) | 0,1667 | 0,1 | Yes | ns |
|  |  |  |  |  |
| Data analyzed |  |  |  |  |
| Sample size, column A | 3 |  |  |  |
| Sample size, column B | 3 |  |  |  |
|  |  |  |  |  |
|  |  |  |  |  |
| **Table Analyzed** | **gst-38** |  |  |  |
| Column B | asna-1(A63V) |  |  |  |
| vs. | vs, |  |  |  |
| Column A | wild-type |  |  |  |
|  |  |  |  |  |
| **Unpaired t test** |  |  |  |  |
| P value | <,001 |  |  |  |
| P value summary | *** |  |  |  |
| Significantly different (P < 0.05)? | Yes |  |  |  |
| One- or two-tailed P value? | Two-tailed |  |  |  |
| t, df | t=12,76, df=4 |  |  |  |
|  |  |  |  |  |
| **How big is the difference?** |  |  |  |  |
| Mean of column A | -0,001 |  |  |  |
| Mean of column B | 0,232 |  |  |  |
| Difference between means (B - A) ± SEM | 0,2330 ± 0,01827 |  |  |  |
| 95% confidence interval | 0,1823 to 0,2837 |  |  |  |
| R squared (eta squared) | 0,976 |  |  |  |
|  |  |  |  |  |
| **F test to compare variances** |  |  |  |  |
| F, DFn, Dfd | 1,580, 2, 2 |  |  |  |
| P value | 0,775 |  |  |  |
| P value summary | ns |  |  |  |
| Significantly different (P < 0.05)? | No |  |  |  |
|  |  |  |  |  |
| **Normality of Residuals** |  |  |  |  |
| Test name | Statistics | P value | Passed normality test (alpha=0,05)? | P value summary |
| Anderson-Darling (A2*) | N too small |  |  |  |
| D'Agostino-Pearson omnibus (K2) | N too small |  |  |  |
| Shapiro-Wilk (W) | 0,9364 | 0,63 | Yes | ns |
| Kolmogorov-Smirnov (distance) | 0,1736 | 0,1 | Yes | ns |
|  |  |  |  |  |
| Data analyzed |  |  |  |  |
| Sample size, column A | 3 |  |  |  |
| Sample size, column B | 3 |  |  |  |

**S10 Fig**. Band intensity quantification of glycosylated vs non-glycosylated SEC-61β (gSEC-61β/ SEC-61β). Bars represent mean ± SD. Statistical significance was determined by the independent two-sample t-test.

|  | ***asna-1(+)*** | ***asna-1(A63V)*** |
| --- | --- | --- |
| **Ratio gSEC-61β/ SEC-61β (%)** | 103,9684227 | 79,54124003 |
|  | 103,7302393 | 90,34448234 |
|  | 92,30133801 | 77,91399721 |

| **Statistical analysis:** |  |  |  |  |
| --- | --- | --- | --- | --- |
|  |  |  |  |  |
| Column B | asna-1(A63V) |  |  |  |
| vs. | vs, |  |  |  |
| Column A | asna-1(+) |  |  |  |
|  |  |  |  |  |
| Unpaired t test |  |  |  |  |
| P value | 0,034 |  |  |  |
| P value summary | * |  |  |  |
| Significantly different (P < 0.05)? | Yes |  |  |  |
| One- or two-tailed P value? | Two-tailed |  |  |  |
| t, df | t=3,175, df=4 |  |  |  |
|  |  |  |  |  |
| How big is the difference? |  |  |  |  |
| Mean of column A | 100 |  |  |  |
| Mean of column B | 82,6 |  |  |  |
| Difference between means (B - A) ± SEM | -17,40 ± 5,481 |  |  |  |
| 95% confidence interval | -32,62 to -2,183 |  |  |  |
| R squared (eta squared) | 0,7159 |  |  |  |
|  |  |  |  |  |
| F test to compare variances |  |  |  |  |
| F, DFn, Dfd | 1,027, 2, 2 |  |  |  |
| P value | 0,987 |  |  |  |
| P value summary | ns |  |  |  |
| Significantly different (P < 0.05)? | No |  |  |  |
|  |  |  |  |  |
| Normality of Residuals |  |  |  |  |
| Test name | Statistics | P value | Passed normality test (alpha=0,05)? | P value summary |
| Anderson-Darling (A2*) | N too small |  |  |  |
| D'Agostino-Pearson omnibus (K2) | N too small |  |  |  |
| Shapiro-Wilk (W) | 0,9317 | 0,593 | Yes | ns |
| Kolmogorov-Smirnov (distance) | 0,2328 | 0,1 | Yes | ns |
|  |  |  |  |  |
| Data analyzed |  |  |  |  |
| Sample size, column A | 3 |  |  |  |
| Sample size, column B | 3 |  |  |  |
