# Supplementary material for: Identification of C. elegans ASNA-1 domains and tissue requirements that differentially influence platinum sensitivity and growth control
Source: PLoS Genet. 2022 Dec 8;18(12):e1010538. doi: 10.1371/journal.pgen.1010538 (PMC9803280; doi:10.1371/journal.pgen.1010538)
Supplement: Attachment — Submitted filename: Response to the Reviewers.pdf [file pgen.1010538.s019.pdf]

Manuscript Title: Non-overlapping requirements of ASNA-1 function for insulin secretion, cisplatin resilience, and growth revealed by genetic analysis of point mutants in *C. elegans*.

Dear Dr Savage-Dunn and Dr Copenhaver,

We would like to thank you and the reviewers for finding our manuscript of interest and raising valuable critical comments. We have carefully considered them and prepared detailed point-by-point response. Moreover, we have revised the title of the manuscript as suggested to emphasize the distinction between this work and our previously published work. The new title of the manuscript: *Identification of ASNA-1 subdomains and tissue requirements that differentially influence platinum sensitivity and growth control.*

As the most important revisions made in the manuscript, we would like to underline:

- Reorganization of the **Figure** numbering in order to improve the flow of the manuscript
- Change in the numbering of the **Supplementary Figures** for them to appear in better order throughout the manuscript
- Transfer of data from **Supplementary Figures** to main **Figures** in order to improve the readability of the data
- Additional DIC microscopy photomicrograph panel in **Figure 2A** as requested by reviewer #1
- Quantification of the L1 arrest caused by pansomatic auxin-mediated ASNA-1 depletion in **Figure 2A** and **Figure 2B** as suggested by reviewer #1 and reviewer #3
- Quantification of the developmental delay caused by intestinal auxin-mediated depletion of ASNA-1 in **Figure 3A** as requested by reviewer #1 and reviewer #3
- Addition of representative images of animals expressing DAF-28::GFP and DAF-16::GFP in **S5 Figure** as requested by reviewer #1 and reviewer #3
- New quantification of oxidized vs reduced ASNA-1^{A63V}::GFP levels in **Figure 6D** as requested by reviewer #2
- New data in **Figure 6E** analyzing levels of oxidized vs reduced ASNA-1^{A63V}::GFP levels after cisplatin exposure as requested by reviewer #2
- New **S13 Figure** showing the full uncropped blot used for quantification of oxidized vs reduced ASNA-1^{A63V}::GFP levels after cisplatin exposure
- New **Table 1** and **S14 Figure** summarizing the concept of the paper

All changes to the manuscript are marked in red. Please note that there is no duplicate publication or submission elsewhere. An earlier version of the manuscript has been posted in BioRxiv (https://www.biorxiv.org/content/10.1101/2022.07.12.499739v1.full). To maximize the reproducibility of research data, we provide a single Excel file containing the source data for each figure in a separate sheet named ‘Source Data’.

Here we provide the detailed point-by-point response to concerns raised by the reviewers:

REVIEWER #1

In the manuscript by Raj et al, the authors use C. elegans to characterize phenotypic effects of different mutations in the essential cytosolic ATPase, ASNA-1 gene. They also examine phenotypes resulting from ASNA-1 being expressed in different tissues. Interestingly, they find that tissue specific expression of ASNA-1 results in different phenotypes – and the same is the case for different ASNA-1 mutations affecting evolutionary conserved amino acids.

This is interesting because it suggests that ASNA-1 can be targeted pharmacologically with a view of specifically influencing certain phenotypes but not others. ASNA-1 is a biomarker for many different diseases. Since ASNA-1 plays an important role in cisplatin resistance and insulin secretion, such targeted intervention has a great potential for example for improved chemotherapy with less side effects.

The paper is well written and easy to read. The data is clearly presented and the rationale underlying the experimental designs are easy to follow. Appropriate controls are included in all experiments.
We would like to thank reviewer #1 for finding our manuscript of interest and providing valuable comments that improved our manuscript.

Minor issues:
In the abstract and in the summary paragraph before results section the performed experiment are mentioned – but what they actually show is not revealed – why not describe the results of the experiments at this point?
We would like to thank the reviewer for the suggestion. We expanded the abstract and added a description of the results.

It would be nice to have a better introduction/description of the Switch I and II domains in the beginning of the paper.
We agree with the suggestion that indeed description of the Switch I and Switch II domains in the Introduction section would be of interest to the readers. We have added such a paragraph to the Introduction section.

Auxin induced degron (AID) – write full first time it is introduced
We have added the full name of the AID abbreviation into the text.

I find it a bit odd that the results section starts with three supplemental figures – it kind of suggests to me that perhaps the order of the first results could be changed a bit?
We agree with the comment. We have reordered the numbering of the figures and renamed **S1 Figure** as **Figure 1**.

The statement "significant delay in development" – lacks proper statistical testing. I am sure the phenotype is fairly clear but without quantification it is an unfortunate choice of word. Growth rate, size, time to first egg lay could easily be measured to allow quantification and statistical testing
We agree with the statement that the 'significant delay in development' statement lacks proper statistical testing. We have now performed a quantitative analysis and the results are presented in **Figure 3**. The data clearly shows that the developmental delay is present after auxin treatment. We are unclear what kind of statistical testing should be performed to substantiate this conclusion.

Structure and function of human proteins – should be plural?
Indeed, the sentence 'structure and function of human proteins' should be in plural and this has been corrected.

…we used the transgenes… - clarify which transgenes?
We clarify now which transgenes have been used for the analysis.

…close contact – rather than closed contact?
We have corrected the grammar in the sentence.

Figure 1 – is the panel with DIC NGM+AUX omitted on purpose?
Yes, the DIC panel was indeed omitted on purpose because we had presented the DIC picture of the same worm in magnification in the third panel. However, now we have added the DIC picture as requested.

It is not clear if the data in Figure 3A and B is just one experiment or a representative example. The results are very clear – but some quantification and statistics would perhaps be appropriate. Same for Figure 4G and H.
We agree with the comment, that this presentation of data did not indicate how many times the experiment was performed. Now we clarified that in the figure legend that experiments were performed in triplicate and statistical testing was performed for cytosolic/nuclear DAF-16:GFP quantification

(**Figure 4A**, previously called **Figure 3A, Figure 5G**, previously called **Figure 4G**). Experiments presented in **Figure 4B** (previously called **Figure 3B)** and **Figure 5H** (previously called **Figure 4G)** were also performed in triplicate, which now is indicated in the appropriate figure legend. However, statistical analysis is not possible on this type of data where all numbers are either 100% (control and *asna-1* single point mutant worms) or 0% (*asna-1* deletion mutant worms). However, to our understanding we believe that interpretation of the results of this particular experiment even without a statistical test will be clear. Moreover, representative images for each graph have been now added and are presented in **S5 Figure**.

…the mutant had no effect on… should be the mutation had no effect on?
Yes, the sentence should be '*the mutation had no effect on*' rather that '*the mutant had no effect on*'. We have corrected the wording in this sentence.

Defect in subcellular – or defective subcellular?
Yes, 'defective subcellular' rather than 'defect in subcellular'. We corrected the wording in this sentence.

I am a little confused about the use of present and past tense in the results section – I think sticking to one makes the paper easier to read. Common practice for the journal should be followed.
We would like to thank the reviewer for his comment on the writing. Indeed, we tried to improve the writing in the manuscript by using the present tense only for data that has been previously showed by our lab and the past tense for the current results showed in this manuscript.

I appreciate that the authors very nicely show that mutations can target only one part (and hence a specific phenotype) of ASNA-1 leaving the other part unaffected. Indeed, as the authors suggest, a similar effect can perhaps be seen with a drug. Perhaps the authors could elaborate on what kind of drug the envision could be used to target fx the Switch I and II domains – and what are their thoughts on doing that without influencing the entire folding of the ASNA-1 protein?
We agree with the comment that more elaboration on the potential drugs that might be used to target the Switch I and Switch II domain would be beneficial. We have added that paragraph to the Discussion section.

Figure S9 Species rather than spices
The writing has been corrected as suggested.

REVIEWER #2
Cisplatin resistance is a major concern in chemotherapy. The authors have published a bolus of work over the past fifteen years demonstrating that ASNA-1 is a bifunctional enzyme that impacts cisplatin toxicity through an ATPase-dependent endoplasmic reticulum tail-anchoring protein (TAP) targeting and insertion activity, but also regulates insulin-insulin-like signaling (IIS) through a separable function. The TAP insertion activity is redox sensitive, with a pair of phylogenetically-conserved cysteines modified to regulate oligomerization and hence activity. While a ASNA-1 loss-of-function mutant is sensitive to cisplatin, it also displays aberrant IIS and is sterile. Importantly, last year this group in a Scientific Reports manuscript described an asna-1 mutant ΔHis164 that prefers the oxidized state and phenocopies the deletion with respect to cisplatin sensitivity, but spares IIS. Since promoting a reduced form of ASNA-1 leads to cisplatin resistance, therapies that target this balance may prove to be impactful for chemotherapeutic success. A better understanding of how this balance is regulated will contribute greatly to devising new therapeutic approaches.
The focus of the current manuscript is two-fold. First, the relative contributions of ASNA-1 in different tissues for growth and reproduction was interrogated through selective depletion using an AID system. Second, a screen of the Million Mutation Project resulted in the identification of a new single site mutant which was then shown to mimic their previous mutant in many ways.
The AID approach used to selectively deplete ASNA-1 for cell-specific "genotype-phenotype" analysis comprised the first half of the manuscript, and while the dataset was extensive, the conclusions were not straightforward. The caveat is that depletion may be incomplete, and there are likely overlapping tissue

requirement as well as cell non-autonomous functions. In addition, it's not clear that depletion occurs uniformly between oxidized tetrameric species, dimers, and monomers – which could be significant, given the redox-balanced output of the protein. These caveats partially countered the novelty and required a nuanced interpretation to make conclusions.
Much of the remainder of the manuscript is very similar in scope and execution to previous work, albeit focused on a different amino acid (A63V) rather than ΔHis164 (from the Scientific Reports paper). This is the largest weakness of the present study. It provides limited new insights, either in TAP insertion, IIS signaling or redox coordination between these two independent functions of ASNA-1. While the approach is generally rigorous and the data is convincing, it does not contribute to an increased appreciation of the underlying molecular processes. With this limitation in mind, the authors show that A63V – like ΔHis164 – exhibits a clear separation of TAP insertion and IIS functions, but without the associated embryonic lethality observed in ΔHis164. This finding may facilitate future ventures by providing a "cleaner" model.

I do have one other specific comment regarding the data. The quantitation of increased quaternary structure in A63V is only marginally convincing based upon the western blots; moreover, the magnitude of the change (400%) is greater than that observed in a previous publication following the addition of 5 mM H2O2 to WT protein (~300%). Also, the western blots (Fig S13) suggest that there is more A63V protein expressed than WT, likely due to the nature of integrated multi-copy, extra-chromosomal arrays. This is important, as expression levels may influence protein oligomerization. It is unfortunate that these experiments were not done by targeting a single copy gene insertion or native ASNA-1 to see whether the A63V mutant exhibits increased quaternary structure under endogenous conditions.
We agree with the comment that there is a discrepancy between the data presented on the western blot and the quantification of mentioned western blots. We have indeed quantified the oxidized and reduced ASNA-1^{A63V}::GFP bands again and found a mistake in the previous calculation. Previously we calculated an increase of ~400%. This has now been corrected and the increase is estimated to be ~170% (**Fig. 6D**) This more accurately represents the western blot data. Moreover, we agree with the comment that transgenic worms produce more ASNA-1^{A63V}::GFP protein in comparison to worms expressing ASNA-1^{+}::GFP. However, the quantification between oxidized vs reduced levels of ASNA-1 was conducted within each strain. We believe that the amount of the transgene in the worm will not influence the ratio between oxidized/reduced amount of ASNA-1.
Moreover, we do agree with the reviewer that targeting a single copy gene insertion or native ASNA-1 would be desirable. However, the detection of endogenous ASNA-1 protein requires an antibody produced against *C. elegans* ASNA-1. A polyclonal antibody of that kind has been produced for us many years ago, but we have run out of it and therefore we were forced to search for an alternative means to detect oxidized ASNA-1 in different experiments. ASNA-1::GFP protein was able to fulfill that requirement on many levels: it allowed us to detect the protein with commercially available GFP antibody and allowed us to differentiate between an oxidized and reduced form of protein. Moreover, expressing the same ASNA-1::GFP transgene in different mutants allowed us to compare results across different genetic backgrounds and drug treatments. This was surely not due to oxidation of the GFP tag moiety since no oxidized version was detected in transgenic worms expressing only GFP (PMID: 33883621). Likewise, we were not able to detect oxidized ASNA-1::GFP when conserved redox-sensitive cysteines in ASNA-1 were mutated to serines (PMID: 33883621).

The authors should consider including H2O2 and cisplatin stimuli to assess whether the mutant responds to oxidative stress in a similar fashion as WT.
As suggested by the reviewer, we assessed the nature of the response to cisplatin of ASNA-1(A63V) protein. We did not observe an increase in oxidized ASNA-1 levels upon cisplatin treatment in ASNA-1^{A63V}::GFP background (**Fig. 6E**).

Please define CAML and VAPB at first appearance – if not full gene name, then function.
Abbreviations for both CAML and VAPB have been defined at their first appearance with their full gene names.

The first sentence in the abstract refers to cisplatin-resistance and type II diabetes, but the Introduction doesn't mention these until the last paragraph and it is unclear whether the context is disease-related or strictly pulled from model organism studies.
We agree with the comment that the first sentence in the abstract refers to the role of ASNA1 in the cisplatin-resistance and type II diabetes. This is followed by the Introduction section describing the role of ASNA1 as a biomarker in treatment response for many diseases. Based on that information, we believe that the relevance of the paper is disease-related and not strictly pulled from model organism studies.

I appreciate the difficulty in being consistent and simultaneously concise when referring to orthologs from different species, but some initial definition of species/gene name would be helpful – it's odd to read "In S. cerevisiae…" prefacing a discussion of GET3 (end of paragraph 3), when the paragraph already contained numerous references to GET proteins in the context of yeast studies.
We agree with the reviewer's suggestion. Indeed, the names for the ASNA-1 orthologs throughout the manuscript might be confusing without a proper explanation at the beginning of the Introduction section. We have now added the name of the species next to the name of the gene in the first sentence of the Introduction section.

Paragraph three of the Introduction is particularly confusing, as it refers to a variety of non-canonical mechanisms without first defining canonical mechanisms.
Paragraph three of the Introduction section indeed refers to a variety of non-canonical mechanisms that are important in the context of ASNA-1 function separation. However, the definition of the canonical mechanism had been provided. Paragraph two of the Introduction section provides information about the most known and best-studied function of ASNA1/GET3 which is a tail-anchored protein insertion.

The final paragraph of the Introduction refers to the findings in the opposite order to which they are presented in the Results.
We have now reorganized the last paragraph in the Introduction section to present the results in the same order as the Results section.

The pan-neuronal ieSi57 TIR1 transgene is introduced twice.
We have corrected that and removed the part where *ieSi57* TIR1 transgene is introduced for the second time.

The strong defect in TAP insertion is claimed to be supported by "three independent tests", but only two are listed.
Indeed, the wording in the text was not correct and the defect in TAP insertion was supported by only two independent tests. We have now corrected this typo.

The integrated arrays containing promoter::ORF::GFP transgenes in VB1428 and GOT159 are not mutant alleles of asna-1. Hence, they should be annotated as svIs56 or rawIs16 in Table S2 rather than asna-1(svIs56) and asna-1(rawIs16).
We have corrected the annotation of the transgenes in **S2 Table.**

It was not clear to me how oxidative stress impacts ANSA-1 activities other than TAP insertion, or whether ASNA-1 even exhibits these activities.
Our previous published work has shown that ASNA-1 promotes DAF-28/insulin secretion (PMID: 17289575) and is required for cisplatin detoxification (PMID: 20966125). Further, the cisplatin and insulin-related phenotypes seen in worm *asna*-1 mutants are seen in cultured mammalian cells and a mouse model. Consistently, expression of the human ASNA1 cDNA rescues both the cisplatin and the IIS phenotypes of *asna-1* mutant worms. Therefore, it is meaningful to extend the observations on the biochemical properties of other orthologs to worm ASNA-1. We note also that on the basis of our work showing that worm ASNA-1 is required for insulin secretion, the work in mice was done to show that mouse ASNA-1 knockdown causes a type2 diabetes phenotype (PMID: 26438609). Given all these findings it is clear that ASNA-1 in worms and ASNA1 in mammals have activities other than that of

TAP insertion. We hope this will answer the reviewer's question about ASNA-1 functions apart from those in TAP targeting.

Regarding the effect of the question of whether oxidative stress impacts ASNA-1 activities: Our recent paper (PMID: 33883621) shows that mutants (*asna-1(ΔHis164)*, *sod-2,* and *mev-1* mutants) which display high levels of oxidized ASNA-1 are cisplatin sensitive (and TAP targeting defective). This shows that the oxidation state matters for its role in cisplatin detoxification.
The ASNA-1 functions other than that for TAP insertion, like the impact of ASNA-1 on insulin signaling and secretion as well as the involvement of ASNA-1 in cisplatin response. Moreover, in a previous publication, we have shown those multiple functions of ASNA-1 can be separated based on the oxidative state of the protein (PMID: 33883621).
However, the evidence of multiple functions of ASNA-1 does not exist only in *C. elegans*. Work on the yeast homolog, GET3, showed that GET3 can act independently of GET1/2, its membrane interacting partner for TAP insertion, to drive different cell biological functions than TAP insertion. This has been also true for the mammalian homolog of ASNA-1, ASNA1/TRC40. To support that statement further, work from our group (under review for another journal; https://www.biorxiv.org/content/10.1101/2022.06.15.496240v1) has identified the ENPL-1/GRP94 protein as a novel ASNA-1 interacting partner and the role of the interaction for insulin secretion.

For example, is ATPase activity required for ASNA's impact on insulin signaling?
Work by Voth *et al.* (2014) (PMID: 25242142) shows that oxidized GET3 acts as a general chaperone, the chaperone activity of GET3 is independent of ATP binding and ATP hydrolysis activities, and that the GET3 chaperone activity is independent of tail-anchored protein targeting. Based on that evidence, we do not believe that ATPase activity would be important for the insulin secretion function of the oligomeric oxidized ASNA-1 protein.

Do mutations that impact ATPase activity but not oligomer formation differentially affect IIS vs. TAP insertion?
Work by Mateja *et al.,* (2009) (PMID: 19675567) shows that several Get3 variants that had lost their capacity to co-immunoprecipitate the TA-protein Sec61beta also were able to retain the high levels of ATPase activity characteristic of a folded protein. In addition, mutants with low relative ATPase activity maintained high levels of TA-protein Sec61beta binding. However, all evidence suggests that mutations that affect the ATPase activity of the protein will also impact the protein fording. Therefore, it is hard to assess the impact of the mutations that only affect ATPase activity but not oligomer formation in the relation to IIS or TAP insertion.

Does the worm ASNA-1 display chaperone or holdase activity under oxidative conditions like GET3? If so, does the A63V mutant enhance this activity? Is the IIS activity that's been shown sensitive to oligomerization, like the chaperone activity?
Our published analysis of the chaperone ENPL-1 (PMID: 33037039) shows that *enpl-1* mutants are also defective in DAF-28/insulin secretion. DAF-28 processing is defective in *enpl-1* mutants and DAF-28 is a client protein of the ENPL-1 chaperone and binds to it via a client binding domain.
We have work under review elsewhere that examines the interaction of ASNA-1 with the chaperone protein ENPL-1 (https://www.biorxiv.org/content/10.1101/2022.06.15.496240v1). Here we showed that ASNA-1 binds to ENPL-1 in ASI neurons – the neuron pair from which DAF-28/insulin is secreted. ENPL-1 preferentially binds to oxidized ASNA-1. Further, the binding between the two proteins is dependent on DAF-28 and evidence shows that it is the pro-DAF-28 form that is required for the binding between ASNA-1 and ENPL-1. The binding of ASNA-1 to ENPL-1 occurs independently of the client binding domain thru which DAF-28 binds the ENPL-1 chaperone. This indicates that ASNA-1 is not a client protein for ENPL-1 and might act as its co-chaperone. Lastly in this work, we show that ASNA-1 can be also found in the Golgi, and the Golgi localization of ASNA-1 is perturbed in *enpl-1* mutants. This line of work provides substantial evidence to support the notion that ASNA-1 displays a chaperone activity and this activity occur in the Golgi and not the ER (where it is acting for TAP targeting). We do not think that the A63V mutant enhances this activity since we do not see increased DAF-28/insulin

secretion in the A63V mutants. Moreover, our data indicates that IIS activity is indeed sensitive to oligomerization since the interaction between ASNA-1 and ENPL-1 increases with elevated ROS levels.

Our working model for ASNA-1 function is that (i) in intestinal cells (and likely elsewhere), the reduced form of ASNA-1 works via WRB-1 at the ER membrane to promote SEC-61β insertion. (ii) In ASI neurons the oxidized form of ASNA-1 works via ENPL-1 in Golgi to promote DAF-28/insulin secretion. These findings underscore the notion of separable functions of the ASNA-1 protein.

Why is so much of the tubulin apparently running at a higher MW selectively in the A63V samples? Is there a known reason why the tubulin controls are apparently always less abundant in samples where ASNA-1 is oxidized (and tetrameric)? This can be noted in the publication from last year, as well, so it's a consistent observation.
In all four experiments, we did not see any discrepancy between the molecular weight of tubulin in ASNA-1^{A63V}::GFP in comparison to ASNA-1::GFP samples (**Figure 6** and **S11 Figure**). The reduced levels of tubulin in non-reduced SDS-PAGE might be due to the fact that the tubulin protein itself is oxidized (PMID: 24215946). However, we do not know how this affects the levels of oxidized tubulin.

It seems important to present a model. Partial inhibitory effects caused by a shift in the ratio of red:ox ASNA-1 impact cisplatin sensitivity, with basement (and perhaps ceiling) effects observed in the complete lf mutant. Some of the concepts discussed here are not intuitive, and a picture is worth 1000 words.
We agree that a summary of the concepts presented in the paper would make it easier for the reader to summarize the data. We have prepared a new **Table 1** and **S14 Figure** that show the most important differences between the loss of function mutants and single point mutants presented in the paper.

The conclusions might be strengthened by employing roGFP to assess redox homeostasis. IMHO, ASNA-1 activity may be part of a compensatory response to oxidative stress? And so the redox status may be altered in response to cisplatin in the mutant. Regardless, this approach would provide additional mechanistic insight to the role of ASNA-1 is modulating cisplatin toxicity.
We would like to thank the reviewer for that suggestion. The idea of ASNA-1 activity being a part of a compensatory response to oxidative stress is indeed interesting and it is something we will consider in the design of our future experiments.

REVIEWER #3
Title:Non-overlapping requirements of ASNA-1 function for insulin secretion, cisplatin resilience, and growth revealed by genetic analysis of point mutants in C. elegans
Authors: Dorota Raj, Agnieszka Podraza-Farhanieh, Pablo Gallego, Gautam Kao and Peter Naredi

This study is focused on the functional effect of two mutations on the metalloregulated ATPase ASNA-1. ASNA-1 function has already been described and has been related with insulin secretion and cisplatin sensibility in mammalian cells and in C. elegans. The authors analyzed the consequences of specific two-point mutants that separate ASNA-1 cisplatin response function from its role on insulin secretion. They used the C. elegans model as part of their experimental strategy and showed the tissue requirements of ASNA-1 for C. elegans growth and development. The authors also showed that targeting single residues in ASNA-1 affecting the Switch I/II domain function, in comparison to the complete knockdown, decreases cisplatin resistance without compromising other important biological functions. This study is in the logical next step from the authors' previous findings, showing that at least two residues can modify ASNA-1 biological function with respect to cisplatin sensitivity.
We thank the reviewer for finding our work to be interesting and for the positive comments.

Nevertheless, in my opinion there are a number of issues that need to be addressed before this paper can be accepted for publication in PLOS Genetics.

1) There is too much relevant evidence in the supplementary figures that makes the paper difficult to follow. In fact, the Results start with supplementary instead with Figure 1, Perhaps the section "ASNA-1 is broadly expressed in C. elegans", could be reorganized as figure 1.

1) We agree with the reviewers comment about the amount of relevant data presented in the Supplementary Figures. Following the advice of the reviewer, we reordered the numbering of the figures and moved the data in **S1 Figure** to a new **Figure 1**.

2) Figure 1. "Soma-specific ASNA-1 depletion leads to L1 larval arrest" must include images (transfer from supplementary figure) and quantification of L1 vs control.

2) Images for soma-specific ASNA-1 depletion which leads to L1 arrest have been transferred from the Supplementary Figure into the main Figure (**Figure 2**). Quantification of L1 arrest has been included and results are presented in **Figure 2**.

3) Figure 2. Intestine-specific ASNA-1 depletion leads to developmental delay. The figure must include the quantitative analysis of the individuals with developmental delay vs control.

3) We have now performed the statistical analysis of developmental delay caused by auxin treatment and the results are presented in **Figure 3**.

4) Figure 3. The figure must include representative images for each graph "cytoplasmic localized DAF-16::GFP ($n \geq 15$) and adults with secreted DAF-28::GFP in coelomocytes ($n \geq 20$). They must include the number of independent assays and statistical analyses must be done.

5) Figure 4. The figure must include representative images for graphs G and H. They must include the number of independent assays and statistical analyses must be done

4 and 5) Representative images for each graph have been now added and are presented in **Supplementary Figure S5**. We clarified in the Figure legend that experiments were performed in triplicate and statistical testing was performed for cytosolic/nuclear DAF-16:GFP quantification (**Figure 4A**, previously called **Figure 3A, Figure 5G**, previously called **Figure 4G**). Experiments presented in **Figure 4B** (previously called **Figure 3B)** and **Figure 5H** (previously called **Figure 4G)** were also performed in triplicate, which now is indicated in the appropriate Figure legend. However, statistical analysis is not possible on this type of data where all numbers are either 100% (control and *asna-1* single point mutant worms) or 0% (*asna-1* deletion mutant worms). However, we believe that the interpretation of the results of this particular experiment even without a statistical test will be clear. Moreover, representative images for each graph have been now added and are presented in **S5 Figure**.

6) The discussion needs to emphasize the relevance of the findings in the context of cisplatin resistance in cancer therapy.

6) We agree with the comment that the importance of the finding should be discussed in the context of cisplatin resistance in cancer therapy. This section has been added to the Discussion.

We would like to thank all the reviewers for raising many interesting and valid points that have improved the manuscript. We hope that our response sheds light on all of the conceptual as well as methodological concerns pointed out by the reviewers.

Yours sincerely,

Dorota Raj Peter Naredi Gautam Kao
