# Supplementary material for: Identification of C. elegans ASNA-1 domains and tissue requirements that differentially influence platinum sensitivity and growth control
Source: PLoS Genet. 2022 Dec 8;18(12):e1010538. doi: 10.1371/journal.pgen.1010538 (PMC9803280; doi:10.1371/journal.pgen.1010538)
Supplement: Attachment — Submitted filename: PLoSG_reveiw_0922.docx [file pgen.1010538.s018.docx]

Cisplatin resistance is a major concern in chemotherapy. The authors have published a bolus of work over the past fifteen years demonstrating that ASNA-1 is a bifunctional enzyme that impacts cisplatin toxicity through an ATPase-dependent endoplasmic reticulum tail-anchoring protein (TAP) targeting and insertion activity, but also regulates insulin-insulin-like signaling (IIS) through a separable function. The TAP insertion activity is redox sensitive, with a pair of phylogenetically-conserved cysteines modified to regulate oligomerization and hence activity. While a ASNA-1 loss-of-function mutant is sensitive to cisplatin, it also displays aberrant IIS and is sterile. Importantly, last year this group in a Scientific Reports manuscript described an *asna-1* mutant *ΔHis164* that prefers the oxidized state and phenocopies the deletion with respect to cisplatin sensitivity, but spares IIS. Since promoting a reduced form of ASNA-1 leads to cisplatin resistance, therapies that target this balance may prove to be impactful for chemotherapeutic success. A better understanding of how this balance is regulated will contribute greatly to devising new therapeutic approaches.

The focus of the current manuscript is two-fold. First, the relative contributions of ASNA-1 in different tissues for growth and reproduction was interrogated through selective depletion using an AID system. Second, a screen of the Million Mutation Project resulted in the identification of a new single site mutant which was then shown to mimic their previous mutant in many ways.

The AID approach used to selectively deplete ASNA-1 for cell-specific “genotype-phenotype” analysis comprised the first half of the manuscript, and while the dataset was extensive, the conclusions were not straightforward. The caveat is that depletion may be incomplete, and there are likely overlapping tissue requirement as well as cell non-autonomous functions. In addition, it’s not clear that depletion occurs uniformly between oxidized tetrameric species, dimers, and monomers – which could be significant, given the redox-balanced output of the protein. These caveats partially countered the novelty and required a nuanced interpretation to make conclusions.

Much of the remainder of the manuscript is very similar in scope and execution to previous work, albeit focused on a different amino acid (*A63V*) rather than *ΔHis164* (from the Scientific Reports paper). This is the largest weakness of the present study. It provides limited new insights, either in TAP insertion, IIS signaling or redox coordination between these two independent functions of ASNA-1. While the approach is generally rigorous and the data is convincing, it does not contribute to an increased appreciation of the underlying molecular processes. With this limitation in mind, the authors show that *A63V* – like *ΔHis164* – exhibits a clear separation of TAP insertion and IIS functions, but without the associated embryonic lethality observed in *ΔHis164*. This finding may facilitate future ventures by providing a “cleaner” model.

I do have one other specific comment regarding the data. The quantitation of increased quaternary structure in A63V is only marginally convincing based upon the western blots; moreover, the magnitude of the change (400%) is greater than that observed in a previous publication following the addition of 5 mM H2O2 to WT protein (~300%). Also, the western blots (Fig S13) suggest that there is more A63V protein expressed than WT, likely due to the nature of integrated multi-copy, extra-chromosomal arrays. This is important, as expression levels may influence protein oligomerization. It is unfortunate that these experiments were not done by targeting a single copy gene insertion or native ASNA-1 to see whether the A63V mutant exhibits increased quaternary structure under endogenous conditions. The authors should consider including H2O2 and cisplatin stimuli to assess whether the mutant responds to oxidative stress in a similar fashion as WT.

Overall, the manuscript is well written, the data transparency is commendable, and the approach and data itself are generally sound. However, the likely impact on the field is more uncertain. I’ve listed some minor concerns below, some cosmetic, some more substantial, and some that are simply questions that I felt could be addressed more clearly in the text.

1. Please define CAML and VAPB at first appearance – if not full gene name, then function.
2. The first sentence in the abstract refers to cisplatin-resistance and type II diabetes, but the Introduction doesn’t mention these until the last paragraph and it is unclear whether the context is disease-related or strictly pulled from model organism studies.
3. I appreciate the difficulty in being consistent and simultaneously concise when referring to orthologs from different species, but some initial definition of species/gene name would be helpful – it’s odd to read “In *S. cerevisiae*…” prefacing a discussion of GET3 (end of paragraph 3), when the paragraph already contained numerous references to GET proteins in the context of yeast studies.
4. Paragraph three of the Introduction is particularly confusing, as it refers to a variety of non-canonical mechanisms without first defining canonical mechanisms.
5. The final paragraph of the Introduction refers to the findings in the opposite order to which they are presented in the Results.
6. The pan-neuronal ieSi57 TIR1 transgene is introduced twice.
7. The strong defect in TAP insertion is claimed to be supported by “three independent tests”, but only two are listed.
8. The integrated arrays containing promoter::ORF::GFP transgenes in VB1428 and GOT159 are not mutant alleles of *asna-1*. Hence they should be annotated as svIs56 or rawIs16 in Table S2 rather than asna-1(svIs56) and asna-1(rawIs16).
9. It was not clear to me how oxidative stress impacts ANSA-1 activities other than TAP insertion, or whether ASNA-1 even exhibits these activities. For example, is ATPase activity required for ASNA’s impact on insulin signaling? Do mutations that impact ATPase activity but not oligomer formation differentially affect IIS vs. TAP insertion? Does the worm ASNA-1 display chaperone or holdase activity under oxidative conditions like GET3? If so, does the A63V mutant enhance this activity? Is the IIS activity that’s been shown sensitive to oligomerization, like the chaperone activity? Given the complexity of the system, it may help to be more explicit.
10. Why is so much of the tubulin apparently running at a higher MW selectively in the A63V samples? Is there a known reason why the tubulin controls are apparently always less abundant in samples where ASNA-1 is oxidized (and tetrameric)? This can be noted in the publication from last year, as well, so it’s a consistent observation.
11. It seems important to present a model. Partial inhibitory effects caused by a shift in the ratio of red:ox ASNA-1 impact cisplatin sensitivity, with basement (and perhaps ceiling) effects observed in the complete lf mutant. Some of the concepts discussed here are not intuitive, and a picture is worth 1000 words.
12. The conclusions might be strengthened by employing roGFP to assess redox homeostasis. IMHO, ASNA-1 activity may be part of a compensatory response to oxidative stress? And so the redox status may be altered in response to cisplatin in the mutant. Regardless, this approach would provide additional mechanistic insight to the role of ASNA-1 is modulating cisplatin toxicity.
